# Loss of dysbindin-1 in excitatory neurons in mice impacts NMDAR-dependent behaviors, neuronal morphology and synaptic transmission in the ventral hippocampus

**DOI:** 10.1038/s41598-024-65566-4

**Published:** 2024-07-02

**Authors:** Sanjeev K. Bhardwaj, Moushumi Nath, Tak Pan Wong, Lalit K. Srivastava

**Affiliations:** 1 Douglas Hospital Research Centre, Douglas Mental Health University Institute, 6875 LaSalle Boulevard, Montreal, QC H4H 1R3 Canada; 2 Department of Psychiatry, McGill University, Montreal, QC Canada; 3 Integrated Programme in Neuroscience, McGill University, Montreal, QC Canada

**Keywords:** Schizophrenia, DTNBP-1, Dysbindin, Animal model, Conditional knockout, Glutamate, Hippocampus, Behavior, Molecular biology, Neuroscience

## Abstract

Dysbindin-1, a protein encoded by the schizophrenia susceptibility gene* DTNBP1*, is reduced in the hippocampus of schizophrenia patients. It is expressed in various cellular populations of the brain and implicated in dopaminergic and glutamatergic transmission. To investigate the impact of reduced dysbindin-1 in excitatory cells on hippocampal-associated behaviors and synaptic transmission, we developed a conditional knockout mouse model with deletion of dysbindin-1 gene in CaMKIIα expressing cells. We found that dysbindin-1 reduction in CaMKII expressing cells resulted in impaired spatial and social memories, and attenuation of the effects of glutamate N-methyl-d-asparate receptor (NMDAR) antagonist MK801 on locomotor activity and prepulse inhibition of startle (PPI). Dysbindin-1 deficiency in CaMKII expressing cells also resulted in reduced protein levels of NMDAR subunit GluN1 and GluN2B. These changes were associated with increased expression of immature dendritic spines in basiliar dendrites and abnormalities in excitatory synaptic transmission in the ventral hippocampus. These results highlight the functional relevance of dysbindin-1 in excitatory cells and its implication in schizophrenia-related pathologies.

## Introduction

Dysbindin-1, a protein with key functions in intracellular protein trafficking and synaptic transmission in rodents, is encoded by the dystrobrevin-binding protein 1 (*DTNBP1*) gene^1,2^. The human ortholog *DTNBP1* is reported to be associated with schizophrenia, responses to antipsychotic drugs as well as cognitive abilities in healthy individuals^3–5^. Even though *DTNBP-1* does not figure in the list of top candidate genes in genome-wide association studies in schizophrenia (Schizophrenia Working Group of the Psychiatric Genomics, 2014), post-mortem investigations have revealed significantly lower expression of dysbindin-1 mRNA and protein in the prefrontal cortex (PFC) and hippocampus tissues of schizophrenia patients^2,6^.

It has been suggested that dysbindin-1 is involved in cognitive deficits^1^, by regulating spine morphology, neurotransmitter release and synaptic plasticity^7,8^. Dysbindin-1 has a well-defined function in protein trafficking in the endosomal lysosomal system, where it binds to proteins in a complex called the biogenesis of lysosome related organelle complex-1 (BLOC-1)^9,10^. In the brain, dysbindin-1 is expressed both pre- and post-synaptically in various neuronal subtypes, as well as in non-neuronal cells, with a significant expression observed in the dopaminergic rich region of the substantia nigra, glutamatergic neurons in the hippocampal formation and PFC and GABAergic neurons of the caudate-putamen^11,12^. Accordingly, there is increasing interest in determining the role of dysbindin-1 in different cellular populations as they pertain to behavioral phenotypes associated with schizophrenia.

Consistent with the expression patterns, studies in Sandy (sdy) mice with a global loss of function mutation in dysbindin-1 show disruptions in glutamatergic, dopaminergic and GABAergic transmission in the PFC and the hippocampus^13–15^. In addition to synaptic alterations, sdy mice also show behavioral abnormalities often associated with schizophrenia, such as deficits in working memory, social interaction, and dopamine (DA)-dependent behavior^13,16^.

Given that dysbindin-1 is ubiquitously expressed, it is possible that the reported effects in Sdy mice are due to dysbindin-1 deficiency in multiple neuronal/non neuronal cell types and brain regions. For example, by conditionally deleting dysbindin-1 gene in DAergic neurons, we showed that a subset of behaviors reported in sdy global mutants are due to the loss of dysbindin-1 in DA neurons^13^. Similarly, another study highlighted a differential effect of dysbindin-1 gene deletion in astrocytes^17^.

In order to assess the role of dysbindin-1 in excitatory neurons, in this study we developed a conditional knockout model with deletion of dysbindin-1 gene in putative excitatory glutamatergic neurons by crossing floxed dysbindin-1 mice with mice expressing cre-recombinase in CaMKIIα expressing neurons (CaMKIIα-cre mice). We found that dysbindin-1 deficiency in CaMKIIα cells results in impaired spatial learning and memory as well as NMDA receptor antagonist MK801-induced locomotion and prepulse inhibition of acoustic startle (PPI). To better understand the mechanisms underlying the behavioral changes, we focused on the ventral hippocampus. The ventral hippocampus has been frequently implicated as a core hub in schizophrenia circuit malfunction^18,19^, and contributes to spatial processing^20^, working memory^21^ and prepulse inhibition^22,23^. We found reduced NMDA-receptor subunit GluN1 and GluN-2B expression, increase in immature dendritic spines on ventral CA1 neurons, and decreased frequency but increased amplitude of excitatory inputs onto ventral CA1 cells. These results complement studies highlighting differential roles of dysbindin-1 in neuronal subtypes and may contribute to a better understanding of its role in schizophrenia etiopathology.

## Methods

### Animals

All methods were performed in accordance with relevant institutional guidelines and regulations. The experiments, approved by the Animal Care and Use Committee of the Douglas Hospital Research Centre, were in compliance with the Canadian Council of Animal Care and ARRIVE guidelines. The procedure for generating mutant animals was adopted as described in our recent report^13^. Briefly, homozygous dysbindin-1floxed mice (dys^loxP/loxP^) were produced at McGill University Transgenic Core Facility by in-vitro fertilization of C57BL/6 female mice with cryo preserved floxed dysbindin-1 sperm (B6N-Dtnbp1^tm1c(EUCOMM)Hmgu/Tcp^; Canadian Mouse Mutant Repository-Toronto Centre for Phenogenomics, Toronto, Canada). Adult offspring were crossed with wildtype C57BL/6 mice for at least 6 generations and the colony was maintained by breeding of heterozygous parents and maintained at the Neurophenotyping facility of the Douglas Hospital Research Centre animal facility. For the current set of experiments, only homozygous floxed dysbindin (dys^loxP/loxP^) animals were used. Homozygous CaMKIIα-Cre^+/+^ mice (Jax stock #005,359) were originally obtained from The Jackson Laboratory and were subsequently bred and maintained on the same C57BL/6 background. At the first level of breeding, we generated a compound, CaMKII-Cre^+/−^/dys^loxP/wt^ mouse line, by crossing dys^loxP/loxP^ mice with Cam-Cre^+/+^ mice. We then backcrossed this compound, CaMKII-Cre^+/−^/dys^loxP/wt^ mice with dys^loxP/loxP^ mice to create CaMKII-positive excitatory neuron specific dysbindin-1 conditional knockout mice (cKO; CaMKII-Cre^+/−^/dys^loxP/loxP^). This breeding step also generated CaMKII-Cre^−/−^/dys^loxP/loxP^ mouse line which were used as experimental controls. At 3 weeks of age, pups were weaned based on sex independent of genotype, and tagged. Mice were housed 4–5 per cage, enriched with loose paper strands such as Enviro-Dri, NESTLET, corn cob bedding and cages were changed on a weekly schedule. They were housed in a temperature and humidity-controlled room with a 12 h light–dark cycle and were given ad libitum access to food and water. All experiments were performed on adult mice of both sexes (8–14 weeks of age at the beginning of the experiments).

### Genotyping and PCR

The animals were genotyped using a duplex polymerase chain reaction (PCR) procedure on tail DNA samples designed to yield PCR products across the segment of floxed dysbindin-1 in cKO mice. The forward and reverse primer sequences for floxed dysbindin-1 are: GGGCTTGTAGCAGATGCTTAGTG and GCAGACCTCAAACTGCTCTTGTATC, respectively. The genotyping results in a single band PCR product of either 804 base pairs (bp) representing homozygotes or 592 bp representing control animals (both bands are present in the heterozygotes). Similarly, to test the presence of CaMKII Cre, DNA extracted from these animals were reprobed using GCGGTCTGGCAGTAAAAACTATC as forward and GTGAAACAGCATTGCTGTCACTT as reverse primers. A PCR product of 100 bp represents the presence of CaMKII Cre. Accordingly, animals that are positive for both homozygous floxed dysbindin and CaMKII cre are cKO mice, whereas animals positive for homozygous floxed dysbindin but negative for CaMKII cre are littermate controls. Heterozygous floxed showed 2 bands at 804 and 592 bp and were omitted from the study.

### Western blotting to measure protein levels of dysbindin-1 and NMDA receptor subunits

Separate cohorts of experimentally naïve adult animals of both sexes, were used to assess the protein levels of dysbindin-1 or NMDA receptor subunit expressions. The procedures for Western blotting were essentially as described previously^13^. The animals were rapidly decapitated and the PFC, hippocampus, and two other control brain regions striatum (STR) and substantia nigra/ventral tegmental area (SN/VTA) were excised and frozen at -80^0^C until use. For dysbindin-1 expression, tissue was sonicated in lysis buffer (50 mM Tris, pH 7.5 containing EDTA (1 mM), NaCl (150 mM), NP-40 (5%), sodium deoxycholate (0.5%) and protease inhibitor cocktail (Sigma cat # P8340 1:10 dilution) and used as described below. For NMDA receptor immunoblotting, the tissue samples were first fractionated as described by^24^. Briefly, the tissue was homogenized in ice-cold 50 mM Tris and 0.32 M sucrose buffer, centrifuged at 1000 × g for 10 min at 4 °C and the supernatant was then centrifuged at 10,000 × g for 20 min at 4 °C. The pellet was washed and resuspended and diluted in 50 mM Tris–HCl lysis buffer. Protein levels were determined using Bradford reagent (Bio-Rad laboratories). The samples (25 μg protein) were electrophoresed and blot transferred onto nitrocellulose membranes (Hybond ECL, Amersham-Pharmacia Biotech) and incubated with the primary antibody against dysbindin-1 (1:2500 dilution; polyclonal antibody; Proteintech cat # 11,132-1-AP), GluN1 (1:1000 dilution; polyclonal antibody Sigma cat # G8913), GluN2A (1:1000 dilution; monoclonal antibody BD cat # 612,286). Separate samples from PFC and hippocampus were probed for GluN2B antibody (1:1000 dilution; polyclonal, Novus Biologicals cat # NB306-106) and for AMPA receptor antibody GluA1 (1:500 dilution; polyclonal antibody, Upstate cat # 06-306). The blots were washed in TBS-Tween-20, and incubated with horseradish peroxidase (HRP)-conjugated secondary antibodies. The blots were developed using chemiluminescence detection system (PerkinElmer). GluN1 blots were restripped and reprobed with GluN2A antibody again. Similarly, GluA1 blots were restriped and reprobed with GluA2 antibody (1:500 dilution: polyclonal antibody, Millipore cat # AB1768-1). To account for variations in protein loading, all blots were stripped and reprobed with antibody against a housekeeping protein. Dysbindin-1 and NMDAR subunit protein levels were calculated using the Relative optical density (ROD; scored with Image J software), normalized to the respective housekeeping protein. Group-comparisons were analyzed on normalized RODs.

### Immunohistochemistry

Control and cKO mice at P60 were anesthetized with a ketamine/xylazine mixture prior to transcardial perfusion with PBS solution followed by 4% paraformaldehyde fixative. Free-floating 40-μm coronal sections along rostrocaudal extents of the PFC, STR and VH were obtained using a vibratome (VT1200s, Leica). Sections underwent a 30-min antigen retrieval step in 0.3% triton-100 in PBS at 37 °C prior to blocking with 5% normal goat serum for 2 h at room temperature (RT). The sections were incubated with 1:250 dilution of primary antibody (rabbit monoclonal anti-dysbindin antibody: Abcam cat# ab124967), first 2 h at RT followed by 48 h at 4 °C. After washing, the sections were treated with secondary alexaFluor 488 conjugated goat antirabbit antibody (1:1000; LifeTechnologies) for 2 h at RT. Sections were mounted on slides in DAPI containing mounting medium. Images were scanned on a fluorescent microscope (Olympus BX-63). Cell counting was performed on images (20X) with maximum projection using the ImageJ (National Institutes of Health, Bethesda, MD). At least three sections per animal/brain region were examined and averaged per animal.

### Behavioral testing

The studies were conducted on the two genotypes of mice (cKO and littermate control) of both sexes (n = 8–18). All behavioral tests were performed between 9 and 18 h. To maximize the use of animals, the same cohort of animals were used for behavioral tests not involving drugs. The tests were performed in the order starting from a lower to higher stressful condition–spontaneous locomotor activity, anxiety like behavior in elevated plus maze, social interaction, and Morris water maze. Following these tests, same cohort of animals were used for MK-801 induced locomotor activity. Tests were separated by 48 to 72 h. Separate cohort of animals were used for saline and MK801 induced PPI. Videorecorded behaviors were scored by an investigator “blind” to the identity of genotypes.

#### Locomotor activity

The assessment of locomotor activity in “animal models” of schizophrenia as a proxy for positive symptoms is based on its dependence on DAergic system and the observations that schizophrenia patients are hyperresponsive to DA-enhancing psychotomimetic drugs as well as glutamate NMDAR antagonists^25^. Since dysbindin-1 has been implicated in the regulation of both DAergic and glutamatergic neurotransmission, including NMDA receptor function^2^, we planned the following experiments.

The spontaneous locomotor activity was measured as described previously^13,26^ using acrylic activity chambers (AccuScan Instruments, Inc., Columbus, OH, USA) (L × W × H = 17.5 cm × 10 cm × 26 cm) in a dimly lit room. The chambers were equipped with infrared sensors; beam breaks by the animals were used to assess locomotor activity. Animals were placed in the activity boxes where their spontaneous locomotor activity was monitored for 90 min. The total horizontal distance traveled (in 10 min bin) and for the whole session was used in the analysis. Data were collected using the Versamax Software (version 4.0, 2004; AccuScan Instruments, Inc.)

We next assessed locomotor response to glutamate NMDAR antagonist. An earlier procedure described in our lab^13,27^ was followed to test the acute effect of MK-801 in littermate control and cKO mice. Animals were individually placed in the locomotor boxes for 30 min to allow them to habituate. Then they received an injection of vehicle (0.9% sterilized normal saline, 5 ml/kg, i.p.). Thirty minutes later, animals received a single dose of MK-801 (0.25 mg/kg, ip, Sigma, UK) and their locomotor activity was recorded for the next 180 min.

#### Basal and MK-801-induced prepulse inhibition (PPI) of acoustic startle

PPI, the ability of a weak stimulus (e.g., auditory) to inhibit the startle response to a subsequent stronger stimulus, is an operational measure of sensori-motor gating and pre-attentional information processing. Among other neural mechanisms, a prominent role of DAergic and glutamatergic systems in the regulation of PPI is recognized and a deficit in PPI is considered to be a translatable endophenotype of schizophrenia^28^.

A new cohort of animals were assessed for MK-801 induced PPI deficit by a within-subject design method. One-half of randomly selected cKO and littermate control animals were injected with the vehicle while the other half received MK-801 (0.25 mg/kg, i.p.) 30 min before PPI testing. Seventy-two hours later, the animals underwent PPI test again with vehicle and drug administration in a counter-balanced order (i.e., saline animals received MK-801and vice-versa). PPI was measured according to our previously described procedure^26^ in SR-LAB system (SR-LAB, San Diego Instruments) boxes. The animals were habituated to the boxes (background noise of 70 dB) for 5 min before giving of startle noise bursts. Mice were first presented with two pilot startle pulses of 120 dB each (the startle responses to these were not included in the analysis). In subsequent trials, the 120 dB startle pulse was either presented alone or 100 ms after 30 ms prepulses of 6, 9, 12 and 15 dB intensity noise bursts above the background noise. These prepulses were presented five times and varied randomly between the trials (average inter-trial interval 15 s). The percent PPI was calculated by the following formula: [100 − (startle response to prepulse and pulse trials) ÷ (startle response to pulse alone trials) * 100]. To assess potential differences in startle characteristic between groups, acoustic startle response to pulse-alone trials (acoustic startle response, ASR) was also analyzed separately.

#### Anxiety-like behavior

There is significant comorbidity between schizophrenia and anxiety disorders and they are more characteristic of the prodromal period^29^. Rodents have natural preference for closed and dark places over open and lit; in the elevated plus-maze (EPM) test, the proportion of time spent in closed arms can be used as a measure of anxiety-like behavior. Anxiety-like behavior in cKO and control mice was evaluated in an EPM according to our previously described procedure^13^. The apparatus (made up of black-painted wood) consists of a plus-shaped maze with two closed and two open arms (50 cm long × 5 cm wide) raised 70 cm above the ground and placed in a dimly lit room (15 Lux). The height of the closed arm walls was 15 cm. Animals were placed in the central arena and allowed to explore the maze for 5 min. The session was recorded with a video camera positioned above the apparatus and the recordings were analyzed for the number of entries into the open and closed arms as well as time spent in the open/closed arms. The ratio of time spent in the open arms vs. the closed arms was used as an index of anxiety-like response.

#### Spatial learning and memory in Morris water maze (MWM)

Cognitive deficits, attributed to hippocampal-PFC circuits (e.g., executive function, spatial working and declarative memory), are considered the “core” symptoms of schizophrenia^30^. We used the MWM test which is sensitive to glutamatergic transmission and commonly used to measure hippocampus-dependent spatial learning and memory in rodents^31^, to assess the impact of loss of dysbindin-1 in glutamatergic neurons.

Our previously described MWM procedure was followed^13,32^. The water maze, consisting of a circular pool (1.4 m diameter and 36 cm high) with a removable plexiglass platform (10 × 10 cm), was placed in a quiet room (light level: ~ 250 Lux) with various distal visual cues and a ceiling-mounted video recording camera. The platform was hidden by pool water (22–24 °C) made opaque with white non-toxic paint. cKO or control mice were released in different quadrants of the pool in a pseudorandom order and their latency to find the fixed and submerged platform was analyzed. Each animal was given three trials per day with an intertrial interval of 1 h. Each trial lasted for 60 s unless the animal reached the platform before this time. The learning trials were given for five consecutive days; the latency data was averaged for each day. Twenty-four hours after the last trial, spatial memory was assessed in the Probe test of a single trial of 60 s with the platform removed. The time spent in the target quadrant and annulus crossing of the platform location were analyzed. At the end of Probe trials, the platform was raised above the water and the animals’ latency to find the visible platform and swim speed were assessed for potential visual or locomotor impairments.

#### Social interaction

Deficits in social cognition are among the important negative symptoms of schizophrenia^33^ Aspects of social interactions in mice are dependent on hippocampal CA1 and CA2 glutamatergic neurons^34^. Previous findings have reported significant deficits in social interaction in sdy null mutants but not in cKO of dysbindin-1 in DA expressing neurons^13,35^. Here we wanted to assess whether the loss of dysbindin-1 in CaMKII expressing excitatory neurons impacts social behaviors in the cKO mice.

Crawley’s three-chamber method of social approach paradigm as described by us recently^13^ was adopted to assess social preference (SP) and social novelty memory (SNM) in the cKO and control mice. The apparatus consists of polycarbonate boxes (60 × 40 × 40 cm) with partitions to create three chambers (each 20 × 40 × 40 cm) placed in a dimly lit quiet room (15 Lux) with a ceiling-mounted video camera to record behavior. The partitions of the box have openings that allow the animal to move freely from one chamber to another. Each side chamber contained an upside-down wire mesh cup with perforated walls and the central chamber is devoid of any objects. A day before the tests, mice were habituated to the box for 20 min with free access to all chambers. On experimental day, the test cKO or control mouse was placed in the central chamber and allowed to explore all chambers for 10 min after which it was returned to the home cage. Next, for SP assessment, a stranger (unfamiliar) mouse (M1) of same strain, sex and age, that had no prior contact with the test mouse, was placed under one of the wire mesh cups in a side chamber (the other side chamber had empty mesh cup). This was followed by reintroduction of the test animal in the central chamber to allow free exploration of all chambers for 5 min. The preferential interaction of the test animal with M1 as compared to the empty wire mesh was taken as a measure of SP or sociability. For SNM measurement, the test animal after SP experiment was returned to the home cage for a retention interval of 10 min. During this time, a second unfamiliar mouse (M2) of same strain, sex and age was placed under the empty wire mesh while the M1 mouse remained under its original mesh. After the retention interval, the test animal was reintroduced into the central chamber and allowed to explore all chambers for 5 min. The preferential interaction of the test animal with M2 relative to M1 was taken as an index of SNM. Social interaction was operationally defined as the time spent in nose contacts or sniffing within a distance of 1 cm or putting at least one paw on the wire mesh with or without the stranger mice. SP was calculated as exploration ratio with the following equation: time spent with the M1 animal/Total time of interaction (M1 + empty mesh) × 100, whereas SNM was calculated as time spent with the second novel animal (M2)/Total time of interaction (M1 + M2) × 100.

### Electrophysiological recordings

Prior studies in sdy null mice have shown in the hippocampus, dysbindin reduction results in increased NMDA but not AMPA-mediated synaptic currents and hence increased LTP, but not LTD^36^. Whereas, in contrast to the hippocampus, dysbindin reduction in the PFC, leads to reduced NMDA-mediated current in sdy null mutant mice^37^. Here, we wanted to assess if the loss of dysbindin-1 in the CaMKII expressing neurons lead to alterations in glutamatergic synaptic transmission in the ventral CA1 hippocampal neurons.

A separate cohort of experimentally adult naïve animals were used for electrophysiological recordings. Adult mice were anaesthetized with isoflurane prior to decapitation. Brains were extracted and quickly submerged in ice-cold carbogenated (95% O_2_ and 5% CO_2_) artificial cerebrospinal fluid (ACSF) containing in (mM): 125 NaCl, 2.5 KCl, 2 CaCl_2_, 2 MgCl_2_, 1.25 NaH_2_PO_4_, 26 NaHCO_3_, and 25 glucose (310–320 mOsm). All reagents were from Sigma-Aldrich (St Louis, MO, USA). Using a vibratome (VT1200s, Leica) ventral hippocampal coronal sections of 300-μm thickness were collected and incubated in carbogenated ACSF for 1 h at 32 C and then for 1 h at room temperature prior to recording. For spontaneous excitatory and inhibitory input recordings, whole-cell recordings on ventral CA1 pyramidal cells were performed^38^ Patch pipettes pulled from borosilicate glass capillaries (World Precision Instruments, Sarasota, FL, USA) were filled with intracellular solution composed of (in mM): 110 K-Gluconate, 17.5 KCl, 10 HEPES, 2 MgCl_2_, 0.5 EGTA and 4 ATP (pH 7.25, 280–290 mOsm). Cells were voltage-clamped at − 60 mV to measure excitatory inputs and at + 10 mV to measure inhibitory inputs. Recordings were amplified using the MultiClamp 700B amplifier (Molecular Devices), filtered at 2 kHz, sampled at 10 kHz, and acquired using the pCLAMP10 program (Molecular Devices). Cells with an access resistance greater than 25 MΩ were omitted. Data were analyzed offline using the Mini Analysis Program 6.0.3 (Synaptosoft, Decatur, GA, USA).

### Golgi-Cox staining for dendritic spines

Several reports show alterations in pyramidal cell dendritic spines in the PFC and the hippocampus of schizophrenia post-mortem samples^39^. Previous studies from knockdown of dysbindin-1 in rat hippocampal cultured neurons have shown that dysbindin is required for the development of dendritic protrusion^8,40^. Here, our aim was to assess whether reduction of dysbindin-1 in excitatory neurons has potential to affect neuronal and dendritic morphology in the ventral hippocampus. A new cohort of experimentally naïve animals were used for Golgi-Cox staining. Adult animals were rapidly decapitated and the brains were extracted and washed with chilled Milli-Q water. Golgi impregnation was performed according to the specifications of the FD Rapid GolgiStain kit (Catalog No. PK401FD; NeuroTechnologies, INC) with the following optimizations: whole brains were treated with silver impregnation for 12 days, cryoprotected with 30% sucrose solution for 72 h, and sectioned (200 μm) in a vibratome in 6% sucrose solution. Brain sections were mounted on gelatin-coated slides, lightly pressed and kept at 4 °C in dark moist container for 7 days, clarified, and then cover slipped using Permount following FD Rapid GolgiStain kit guidelines. Slides were allowed to dry for two weeks before neuron tracing was performed using a Zeiss Observer Z1 MBF Microscope and Neurolucida Software (MBF Bioscience)^41^. Approximately 3 hippocampal vCA1 neurons per mouse were traced, from different sections and locations of the ventral hippocampus. Only neurons with fully visible processes were selected for tracing. Data for spine number, process length, number of nodes, and primary processes were obtained using Neurolucida explorer. Mean values of total basilar dendritic length and spine density was calculated.

### Statistics

Data are presented as mean ± standard error of the mean in each group. Data were analyzed using GraphPad Prism (version 8). To assess potential sex-specific effects, we first analyzed data using two- or three-way analysis of variance with sex as an independent variable (between factor) as suggested by^42^. Where ANOVA did not reveal main effects of sex or genotype x sex interaction, we pooled the data from both the sexes for further analyses using student’s *t*-tests or factorial ANOVA with *Tukey’s* post-hoc test as appropriate.

## Results

### Reduced expression of dysbindin-1 in excitatory neuron-rich brain regions of mice

**Figure 1:**
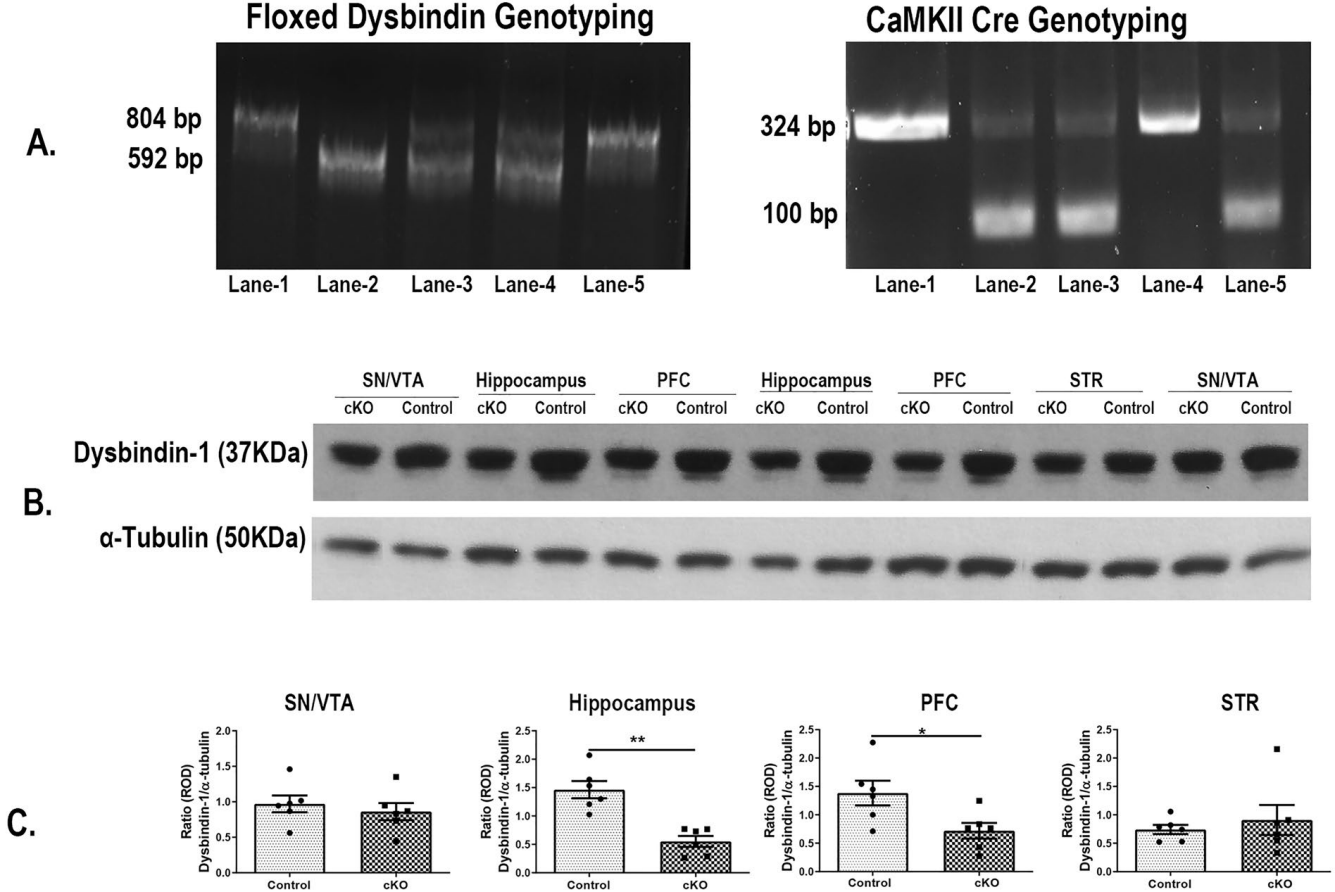
Selective loss of Dysbindin-1 expression in cKO animals: (**A**) Genomic PCR. Homozygous floxed dysbindin-1 showed a band at 804 bp (lanes-1 and -5) while heterozygous floxed showed 2 bands at 804 and 592 (lane-3 and 4). Genotyping product of CaMKII Cre showed the presence of a band around 100 bp. Animal in lane-5 showed the presence of CaMKII cre and homogeneous floxed dysbindin (C1L2) and was used as cKO animals. (**B**) Representative Western blots of dysbindin-1 protein expression in different tissues of control and cKO mice from 2 different mice. (**C**) Quantitation of the Western blot showing a selective reduction of dysbindin-1 protein expression in the PFC and hippocampus of cKO animals. n: male control = 5, female control = 1, male cKO = 5, female cKO = 1. *p = 0.028 vs control and **p < 0.001 vs control.

Figure 1A shows the representative genotyping images of both floxed dysbindin and CaMKII cre mice. As mentioned earlier, animal in lane-1 is control and in lane-5 is cKO.

Unpaired, two-tailed t-test of western blots showed a significant reduction of dysbindin-1 protein in the excitatory neuron rich regions of the hippocampus (t(10) = 5.06, *p* < 0.001) and the PFC (t(10) = 2.57; *p* = 0.028) of cKO mice compared to littermate controls. In contrast, the dysbindin-1 expression was not significantly altered in other brain regions (STR (t(10) = 0.60, *p* = 0.56) and SN/VTA (t(10) = 0.64, *p* = 0.53) (Fig. 1B,C). The raw figures of the western blots are presented in supplementary file.

### Decreased dysbindin-1 immunostaining in cKO animals

**Figure 2:**
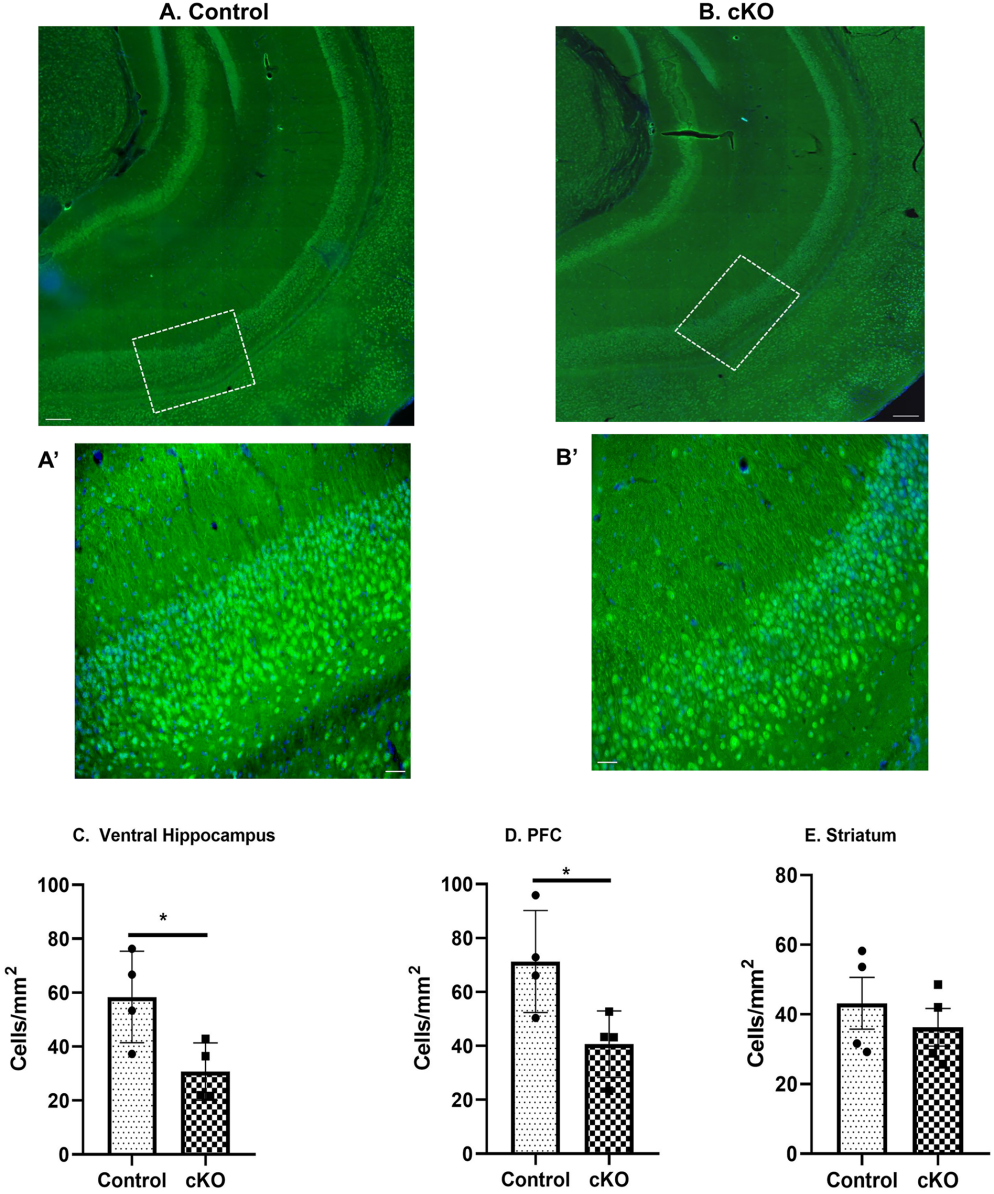
Immunohistochemical staining for dysbindin-1 in the hippocampus of adult control and cKO mice. (**A** and **B**) Representative images of sections from control and cKO mice respectively at 4X. Scale bar: 500 μm. Inset in the figure shows the area from the ventral hippocampus at 20X was used for quantification. (**A’** and **B’**) shows the representative magnified (20X) images obtained from control and cKO animals respectively for quantification. (**C**–**E**) shows the quantification of the dysbindin-1 positive cells from both control and cKO mice at 20X magnification from ventral hippocampus, PFC and STR respectively. Significantly reduced number of dysbindin-1 positive cells was observed from ventral hippocampus and PFC of cKO animals. No significant differences were observed in STR brain region. N = 4 each group. *p = 0.0325 vs control (ventral hippocampus) and *p = 0.0348 vs control (PFC).

To validate our western blot data, we used immunohistochemistry to assess dysbindin-1 deficiency in glutamatergic neurons. We selected sections from glutamatergic neurons rich PFC and ventral hippocampus brain regions along with STR as a control area. Figure 2A,B show the representative images of sections from control and cKO mice respectively at 4X whereas Fig. 2A’,B’ show the representative magnified (20X) images obtained from control and cKO animals respectively for quantification. Two-tailed, unpaired *t*-test result showed a significant decrease in dysbindin-1 staining in ventral hippocampus (t(6) = 2.768; *p* = 0.0325) and PFC (t(6) = 2.717; *p* = 0.0348) of cKO mice compared to control animals (Fig. 2C,D respectively). No significant differences were observed in the STR (t(6) = 0.7513; *p* = 0.4809) (Fig. 2E). Thus, our results from both western blot and immunohistochemistry indicate a reduction of dysbindin-1 protein level in the glutamatergic rich brain areas.

### Behaviors

#### Spontaneous locomotor activity in novel environment

**Figure 3:**
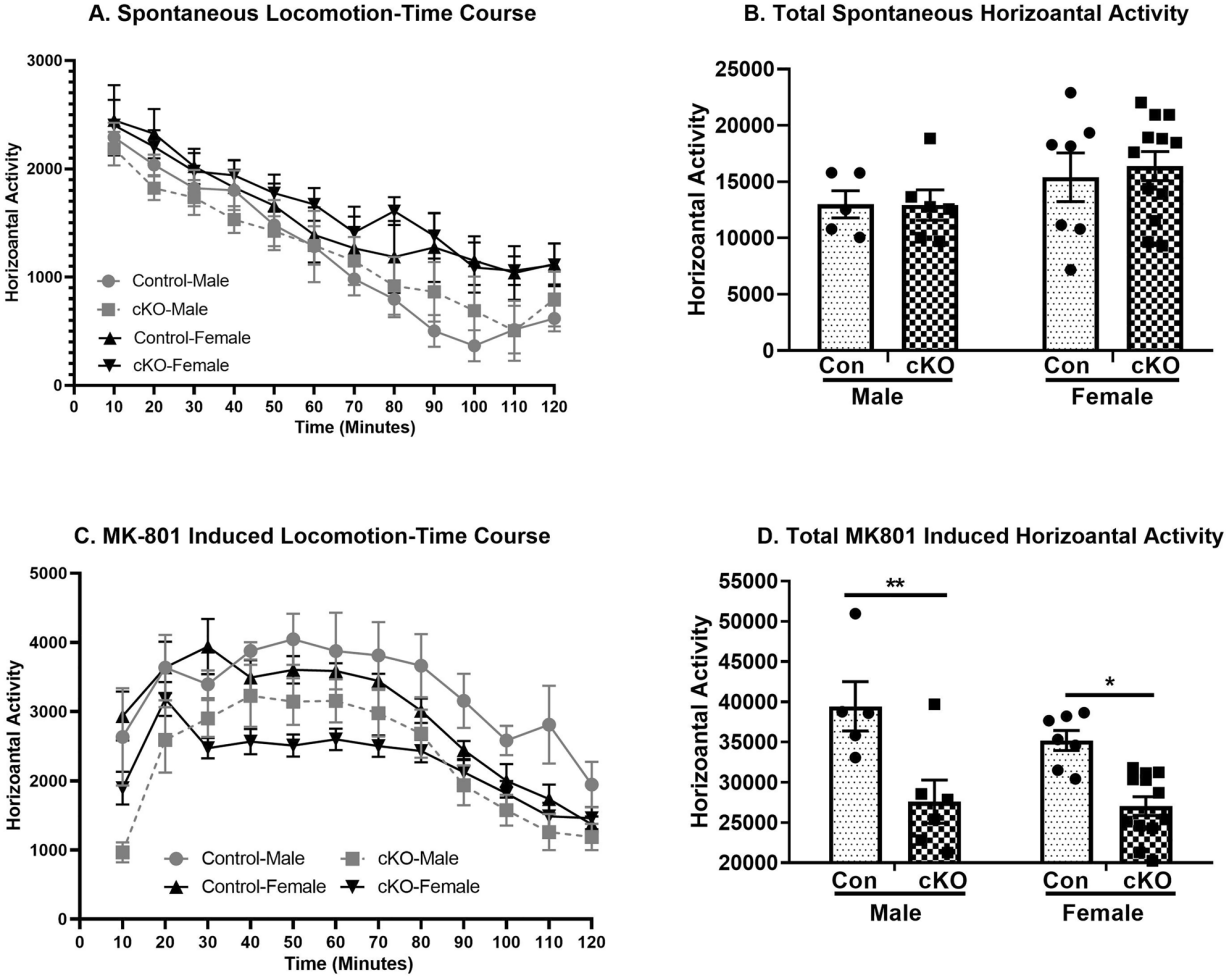
Attenuated MK-801 induced locomotor activity in cKO animals. (**A**) Time-course of spontaneous locomotor activity of control and cKO mice of both the sexes in a novel environment. A 3-way repeated measure ANOVA did not show any significant genotype × sex x time interactions. (**B**) Total spontaneous locomotor activity of both sexes from control (Con) and cKO mice in a novel environment did not show significant differences between control and cKO animals. (**C**) Time-course of acute MK-801 (0.25 mg/kg) induced locomotor activity of control and cKO mice of both the sexes. A 3-way repeated measure ANOVA showed a significant genotype × sex x time interactions. (**D**) Total MK-801 induced locomotor activity from both sexes of control and cKO animals. A 2-way ANOVA showed a significant main effect of genotype but no genotype x sex interactions. However, a planned comparison revealed that MK-801 induced locomotor activity was significantly decreased in both males and females cKO animals compared to respective controls. n: male control = 5, female control = 7, male cKO = 6, female cKO = 12. **p < 0.001 vs control-male and *p = 0.003 vs control-female.

Figure 3A shows that the loss of dysbindin-1 in CaMKII expressing cells in cKO mice had no significant effect on spontaneous locomotor activity. A three-way repeated measure ANOVA on locomotion data over time showed a significant main effect of time (F_11,286_ = 25.65, *p* < 0.001), but no significant effect of genotype (F_1,26_ = 0.07, *p* = 0.79), sex (F_1,26_ = 0.10, *p* = 0.10) and no significant three-way interaction (genotype x sex x time) (F_11,286_ = 0.38, *p* = 0.96). Further analysis was performed on total locomotor score for the whole session (Fig. 3B). A two-way ANOVA showed no significant effects of genotype (F_1,26_ = 0.071, *p* = 0.79), sex (F_1,26_ = 0.10, *p* = 0.10), or genotype x sex (F_1,26_ = 0.096, *p* = 0.75) interaction.

#### MK-801 induced locomotor activity

Figure 3C shows that dysbindin-1 reduction in cKO mice leads to a significant attenuation in locomotor activity following acute MK801 administration. A three-way repeated measure ANOVA on locomotion data over time showed a significant main effect of genotype (F_1,26_ = 27.12, *p* < 0.001), time (F_11,286_ = 32.48, *p* < 0.001) and three-way interaction (genotype x sex x time) (F_11,286_ = 1.92, *p* = 0.036), but no significant main effect of sex (F_1,26_ = 1.56, *p* = 0.22) was observed. Further analysis was performed on the total locomotor activity for the whole session. A two-way ANOVA on MK-801 induced locomotor activity revealed a significant main effect of genotype (F_1,26_ = 27.12, *p* < 0.001), but no significant effect of either sex (F_1,26_ = 1.56, *p* = 0.22) or genotype x sex interactions (F_1,26_ = 0.92, *p* = 0.34). A planned comparison post-hoc analysis revealed that MK-801 induced locomotor activity was significantly lower in both male (*p* < 0.001) and female (*p* = 0.002) cKO mice compared to respective controls (Fig. 3D).

#### MK-801-induced PPI deficit

**Figure 4:**
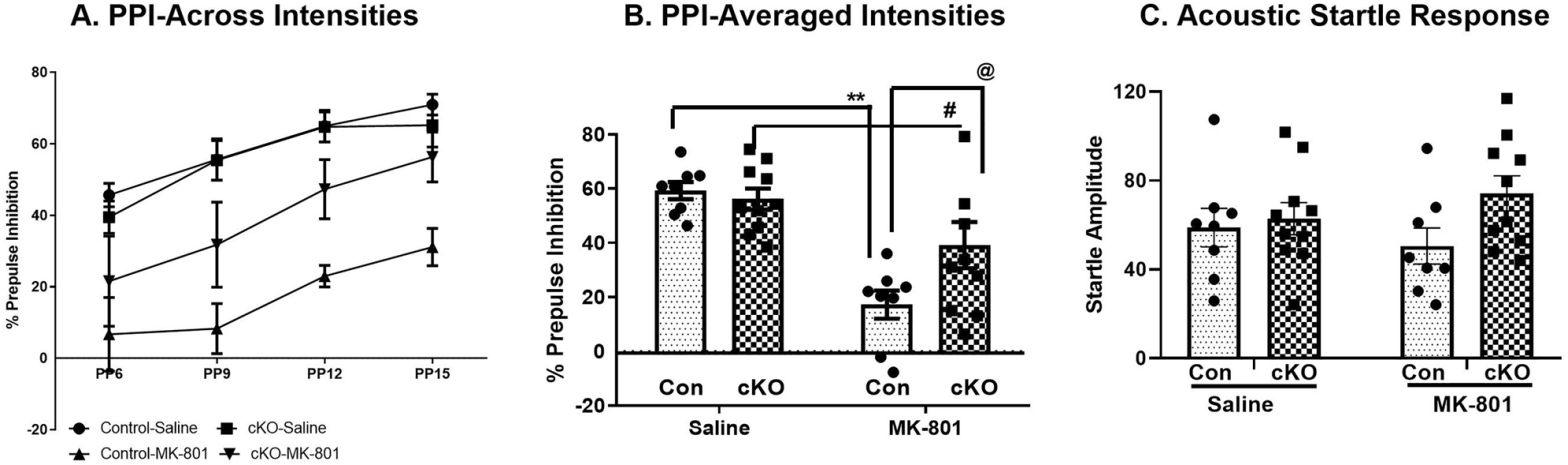
Reduced MK-801-induced PPI deficit in cKO mice. (**A**) The effect of saline and MK-801on PPI disruption across all prepulse intensities (PPs). A 3-way ANOVA showed a significant main effect of drug and PPs but no 3-way interaction. (**B**) The effect of MK-801 on % PPI at the collapsed PPs level. Two-way ANOVA showed a significant genotype x MK-801 interaction. (**C**) Acoustic startle response (ASR) was not significantly different between the groups. n: male control = 4, female control = 4, male cKO = 5, female cKO = 5. **: vs control-Saline, ^#^: vs cKO-saline, ^@^: vs control-MK-801 animals.

Our results show that although basal levels of PPI are comparable in cKO and controls, acute MK-801 administration leads to a significantly smaller PPI deficit in in the cKO mice, compared to the controls. Data on % PPI were first analyzed using a three-way repeated-measure ANOVA with genotype and drug as between factors and prepulses (PPs) as within (repeated measure) factor. It showed a significant effect of PPs (F_3,96_ = 23.13, *p* < 0.001), MK-801 treatment (F_1,96_ = 24.9, *p* < 0.001) and genotype x MK-801 interactions (F_1,96_ = 4.51, *p* = 0.04). There were no other significant 2-way or 3-way interactions [(genotype x PPs (F_3,96_ = 0.48, *p* = 0.69), MK-801 × PPs (F_3,96_ = 0.80, *p* = 0.49)] [genotype x MK-801 × PPs (F_3,96_ = 0.17, *p* = 0.91)] (Fig. 4A). As the analysis did not show any significant interaction with PPs, we collapsed (averaged) all PPs for a further 3-way analysis using sex, genotype and drug as independent variables. This analysis showed a significant main effect of the drug (F_1,28_ = 22.78, *p* < 0.001) and drug x genotype interaction (F_1,28_ = 4.11, *p* = 0.05), but not sex (F_1,28_ = 0.23, *p* = 0.63), genotype (F_1,28_ = 2.33, *p* = 0.13), drug x sex (F_1,28_ = 0.00, *p* = 0.96) or genotype x drug x sex interactions (F_1,28_ = 0.16, *p* = 0.69). Tukey’s post-hoc analysis revealed that in control animals MK-801 administration led to a significant PPI deficit compared to saline injection (*p* < 0.001; Fig. 4B). The cKO mice also showed a significant deficit in PPI following MK-801 treatment compared to cKO-saline animals (*p* = 0.038). However, MK-801-induced PPI deficits were significantly less in cKO mice compared to control mice (29.9% vs 67%; Fig. 3B). Baseline acoustic startle response (ASR) did not differ between the groups (two-way ANOVA: no significant genotype x treatment interaction (F_1,32_ = 1.51, *p* = 0.22; Fig. 4C).

#### Anxiety like behavior in the EPM

**Figure 5:**
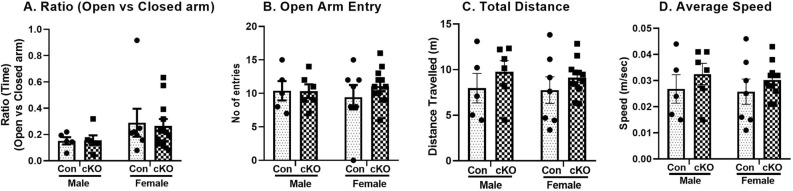
No significant change in anxiety like behavior in control (Con) and cKO animals EPM. (**A**) Ratio of the time spent in open vs. closed arms. (**B**) Number of entries in open arms. (**C**) Total distance travelled and (**D**) Average speed of the mice of both gentypes on the maze were also comparable. n: male control = 5, female control = 7, male cKO = 6, female cKO = 12.

A 2-way ANOVA on the time spent in open arm did not show significant main effect of either genotype (F_1,26_ = 0.018, *p* = 0.89), sex (F_1,26_ = 2.98, *p* = 0.10) or genotype x sex interactions (F_1,26_ = 0.04, *p* = 0.84) (Fig. 5A). Similarly, the analysis on the open arm entry also did not show any significant main effect of either genotype (F_1,26_ = 0.39, *p* = 0.54), sex (F_1,26_ = 0.01, *p* = 0.93) or genotype x sex interactions (F_1,26_ = 0.45, *p* = 0.51) (Fig. 5B). Total distance travelled and average speed of both genotypes of animals in the maze was also comparable (Fig. 5C,D respectively). These data showed that dysbindin-1 reduction in CaMKII expressing cells had no significant effect on the measures of anxiety-related behaviors,

#### Spatial learning and memory

**Figure 6:**
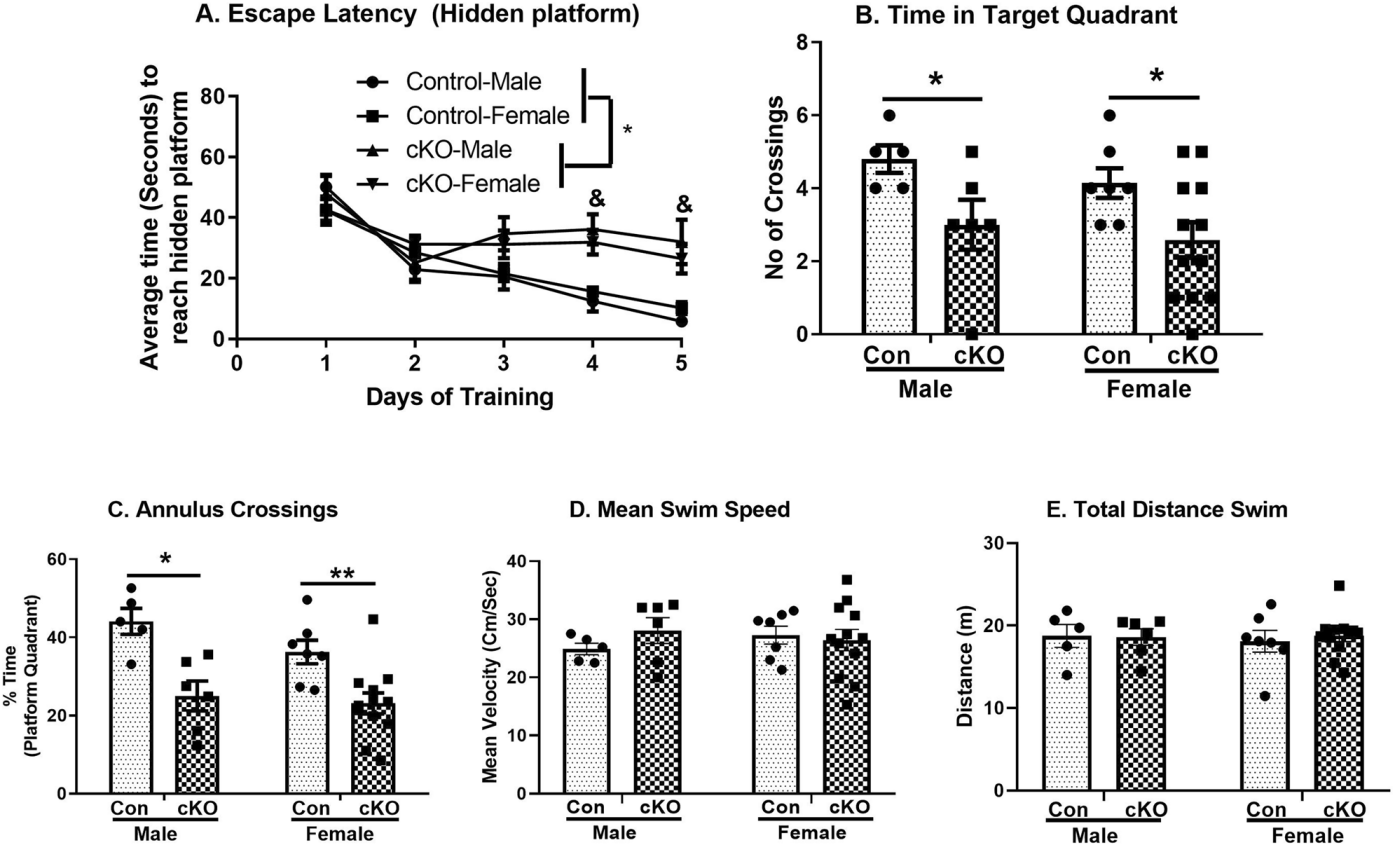
Impaired spatial learning and memory in cKO animals in Morris Water Maze. (**A**) Escape latencies during hidden platform trials. cKO animals have significant deficit in spatial learning compared to controls. In spatial memory task of probe test, both sexes of cKO mice showed significant deficits in the time spent in correct quadrant (**B**) and average annulus crossings (**C**) compared to control (Con) mice. (**D**) Latency to reach visible platform and (**E**) total distance swam were not different between both genotypes. n: male control = 5, female control = 7, male cKO = 6, female cKO = 12. ^&^: *p* < 0.05 vs control-male, *: *p* < 0.05; **: *p* < 0.01.

Our results showed that cKO mice with dysbindin-1 reduction in CaMKII expressing cells have significantly impaired spatial learning and memory in the Morris water maze (Fig. 6A). Latency data across learning trials were analyzed using three-way ANOVA with genotype and sex as independent variables and days of training as repeated measure (Fig. 6A). We found a significant main effect of genotype (F_1,104_ = 10.42, *p* < 0.001), days of training (F_4,104_ = 30.21, *p* < 0.001) and genotype x days interactions (F_4,104_ = 7.25, *p* = 0.001). No main effect of either sex (F_1,104_ = 0.03, *p* = 0.85), genotype x sex (F_1,104_ = 0.30, *p* = 0.58), sex x days (F_4,104_ = 1.40, *p* = 0.14) or genotype x days x sex interactions (F_4,104_ = 0.49, *p* = 0.74) were observed. Post-hoc *Tukey’s* test showed that male cKO animals have deficits in spatial learning as revealed by a significantly higher latency to reach platform compared to control male animals, on days 4 and 5 (*p* < 0.05). Similarly female cKO animals showed a trend of higher latency to reach hidden platform on day 4 and 5 (*p* = 0.09).

The analysis of spatial memory (probe test) showed a significant main effect of genotype (F_1,26_ = 22.98, *p* < 0.001) but not of sex (F_1,26_ = 2.10, *p* = 0.15) or genotype x sex interactions (F_1,26_ = 0.79, *p* = 0.38). Post-hoc analysis showed that both male and female cKO animals show spatial memory deficits as they spent significantly less time in the target quadrant (*p* = 0.014 and 0.004, respectively; Fig. 6B). Similarly, the analysis of annulus crossings confirms memory deficit in cKO mice with a significant main effect of genotype (F_1,26_ = 9.07, *p* = 0.005). No significant main effect of either sex (F_1,26_ = 0.93, *p* = 0.34) or genotype x sex interactions (F_1,26_ = 0. 046, *p* = 0.83) were observed. Post-hoc analysis revealed that both male (*p* = 0.05) and female (*p* = 0.032), cKO animals have significantly fewer annulus crossings compared to respective controls (Fig. 6C). The mean swim speed to reach visible platform and total distance swam were not significantly different between the two genotypes, indicating that motor and/or visual abilities of the cKO mice are not impaired (Fig. 6D,E respectively).

#### Social interaction

**Figure 7:**
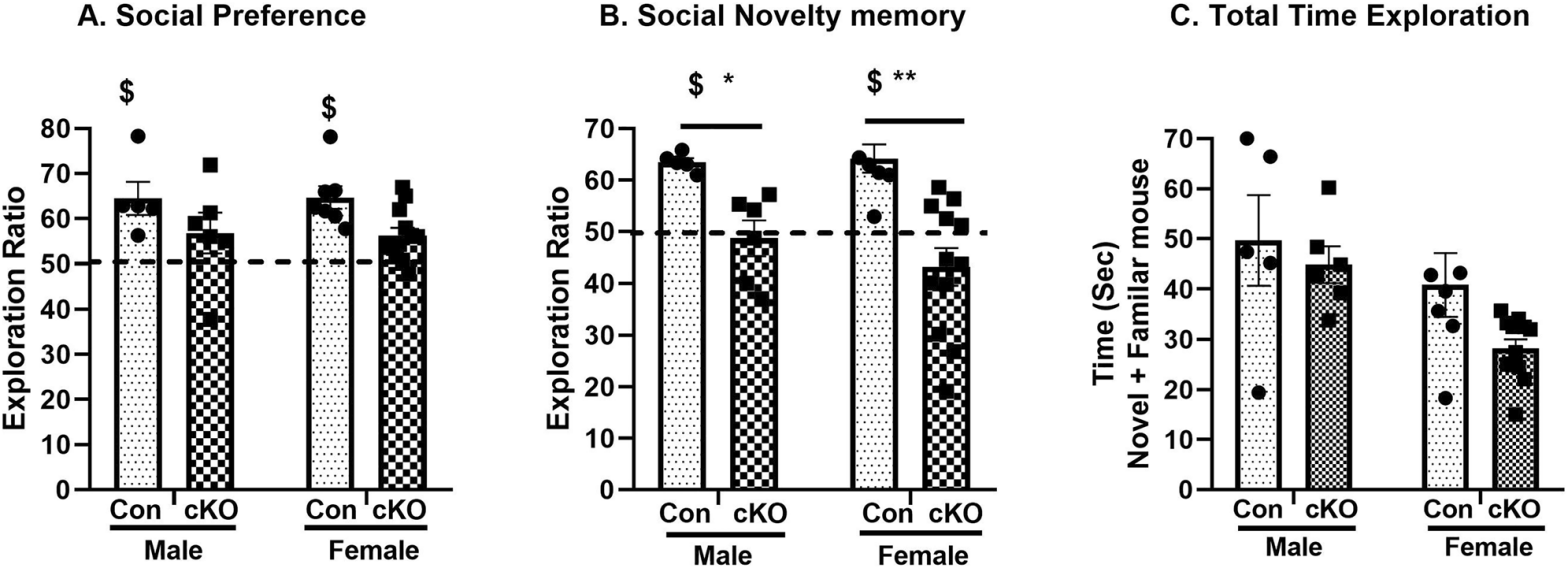
Decreased social memory in cKO animals. (**A**) No significant differences in the social preferences (novel mouse over the empty mesh cage) between the two groups of animals. (**B**) A significant decrease in social novel memory (novel mouse over the familiar mouse) was observed in cKO animals. Only control (Con) animals showed preferences for novel mice in social preference and social novelty memory tasks (one-sample *t*-test) (**C**) No change in the total time of exploration of both the animals was observed. n: male control = 5, female control = 7, male cKO = 6, female cKO = 12. *p = 0.018; **: *p* = 0.003 vs Control, ^$^: *p* < 0.05 compared to chance level, i.e. 50%.

The results from 3-chamber social tests showed that dysbindin-1 reduction in CaMKII expressing cells causes disruptions in social behavior. A 2-way ANOVA of the social preference did not show any significant main effect of either sex (F_1,26_ = 0.03, *p* = 0.95) or genotype x sex interactions (F_1,26_ = 0.14, *p* = 0.90), but a main effect of genotype (F_1,26_ = 7.42, *p* = 0.011) was observed. However, a planned comparison post-hoc analysis did not reveal any further group differences (Fig. 7A). Similarly, the analysis on the social novelty memory (SNM) also showed a significant main effect of genotype (F_1,26_ = 23.10, *p* < 0.001), but no main effect of either sex (F_1,26_ = 0.43, *p* = 0.51) or genotype x sex interactions (F_1,26_ = 0.70, *p* = 0.41). Since a significant main effect of genotype is observed in SNM, a planned comparison post-hoc analysis revealed that both male (*p* = 0.018) and female (*p* < 0.001) cKO animals showed significantly less preference for novel mouse compared to respective controls (Fig. 7B). Further, an analysis of the exploration ratio using one-sample *t*-test (compared to chance level, i.e. 50%), revealed that only control animals (both sexes) showed significant preference for novel mice vs empty mesh cups (in social preference task) and for novel mice vs familiar mice (in social novelty memory task), while cKO animals failed to reach such significance. The total time of exploration by the cKO animals were not significantly different from the controls Fig. 7C).

### Electrophysiological recordings in ventral CA1 pyramidal neurons

**Figure 8:**
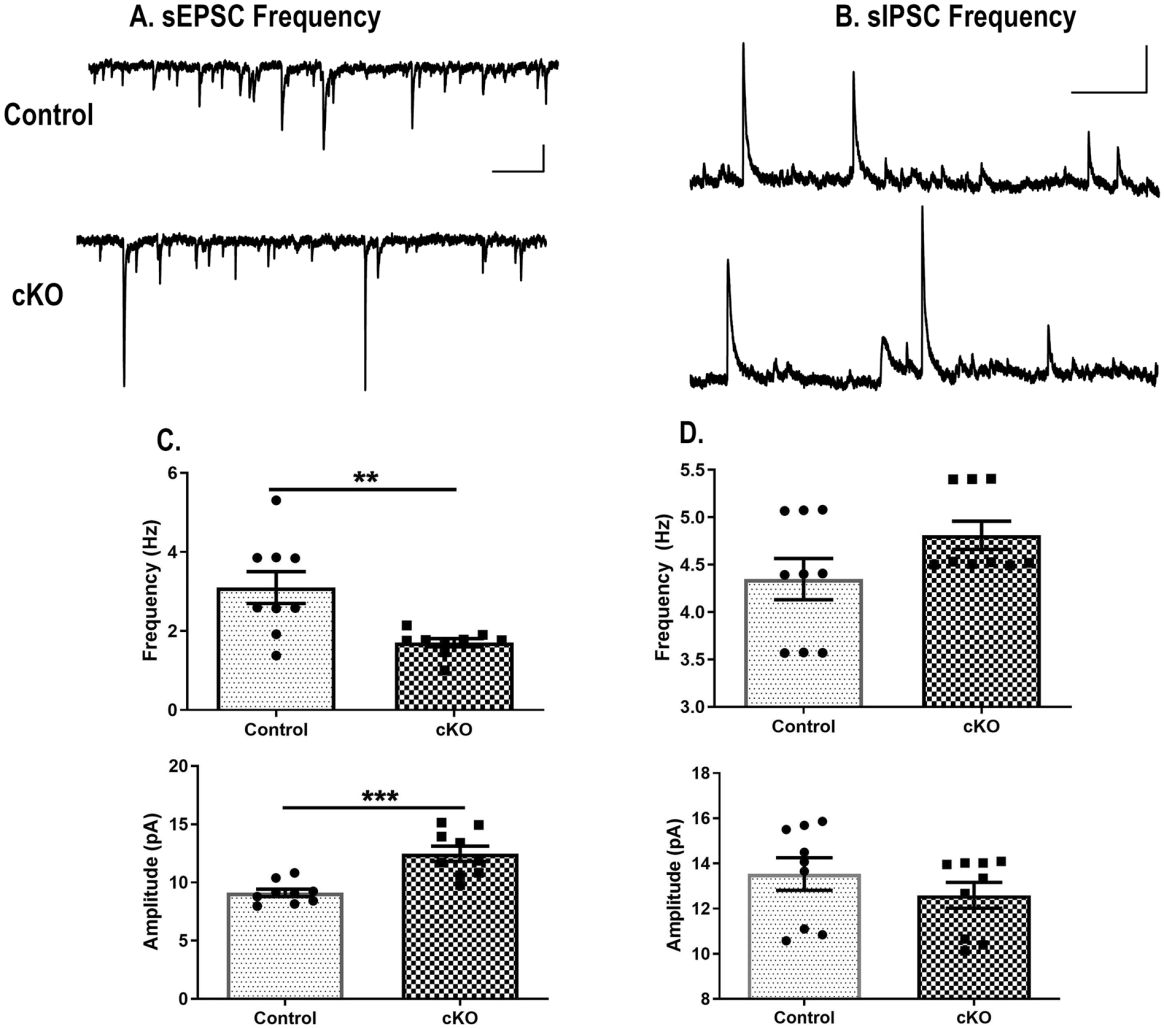
Decreased frequency but larger amplitudes of excitatory inputs in cKO animals. (**A**) Sample sEPSC traces (scale bar: 500 ms, 20pA) and (**B**) sIPSC traces (scale bar: 200 ms, 50pA). (**C**) cKO mice show decreased sEPSC frequency compared to control animals. These sEPSC events are also of larger amplitude compared to control mice. (**D**) No significant differences were observed in sIPSC frequency or amplitude respectively between two genotypes of animals. n: male control = 9, male cKO = 9. **: *p* = 0.004 vs Control, ***: *p* = 0.0003 vs Control.

As our behavioral data did not reveal any sex dimorphic effects, the following experiments were conducted in male animals. Whole-cell recordings of ventral CA1 pyramidal cells revealed a decreased spontaneous excitatory input frequency (sEPSC) in cKO mice (t(16) = 0.004) (Fig. 8A,C top panel). These sEPSCs were also on average of larger amplitude (t(16) = 0.0003) (Fig. 8C lower panel). No significant differences were observed in spontaneous inhibitory input frequency or amplitude (sIPSCs) between the cKO and control genotypes (Fig. 8B,D). These results highlight that conditional ablation of dysbindin-1 in CaMKII-expressing cells interferes with excitatory transmission.

### Golgi-Cox analysis of ventral CA1 pyramidal neurons

**Figure 9:**
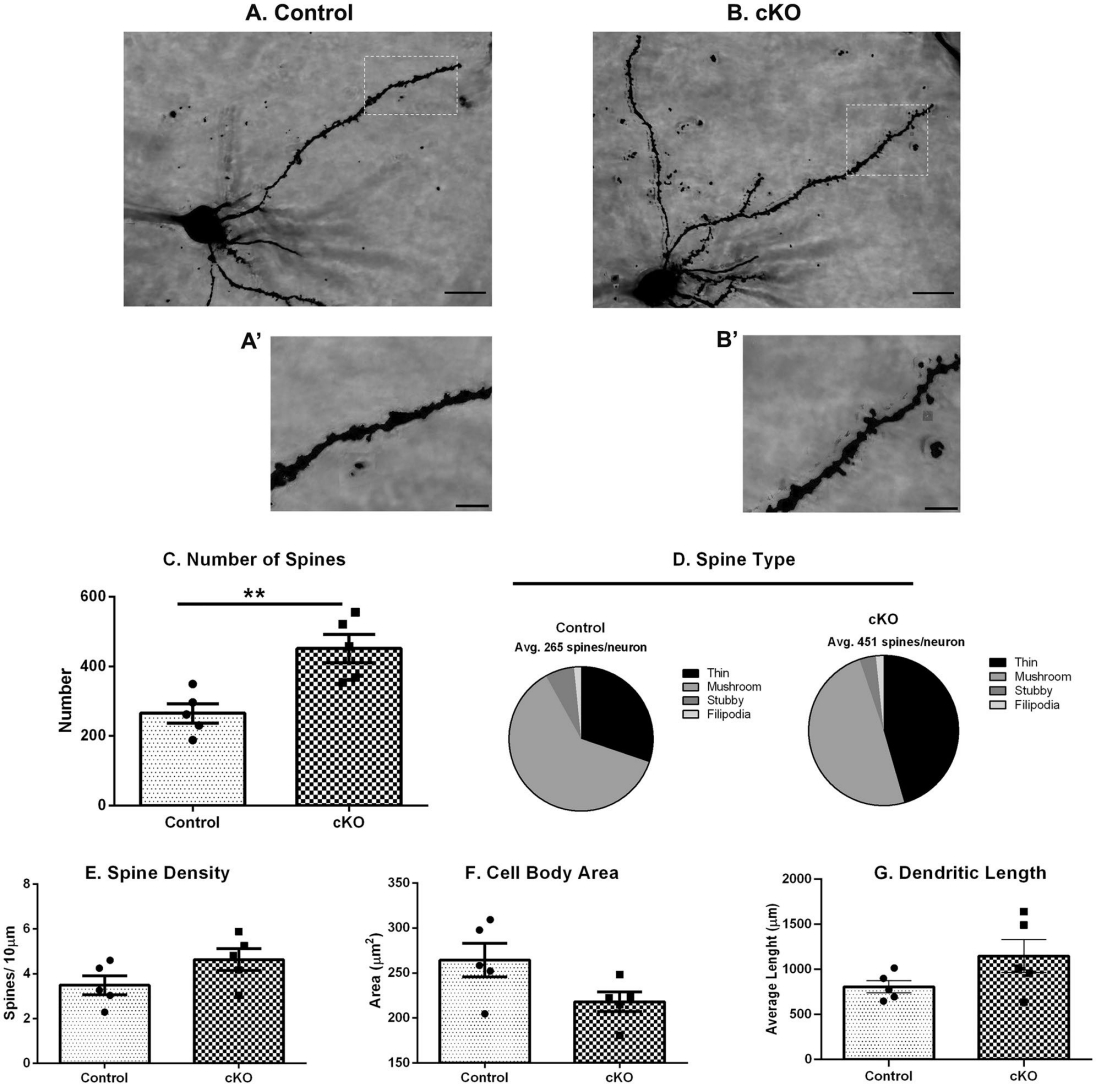
Altered spine morphology in ventral CA1 neurons in cKO animals. (**A** and **B**) Representative images of Golgi-stained vCA1 pyramidal neurons of control and cKO mice at adulthood (Scale bar: 10 μm). Inset shows the area (**A’** and **B’**) from the basilar dendrites at 60X magnification, obtained from control and cKO animals, respectively, (**C**) Significantly increased number of spines are observed in cKO mice. (**D**) Further, analysis showed an increase from 30 to 45% in thin spines in cKO animals. However comparable spine density (**E**), total area of the neuron (**F**) and total dendritic length (**G**) was observed between control and cKO mice. n: male control = 5, male cKO = 5. **: *p* = 0.006 vs Control.

Our results show that Golgi-Cox staining in control and cKO mice indicates that dysbindin-1 deficiency in CaMKII expressing cells is associated with increased dendritic spines in the vCA1 cells. Figure 9A,B show the representative photomicrographs of Golgi-stained vCA1 pyramidal neurons of control as well as cKO mice at adulthood, whereas Fig. 9A’,B’ show the representative magnified (60X) images obtained from control and cKO animals respectively. Analysis of the dendritic spine morphology of ventral CA1 pyramidal cells showed a significant increase in the total number of spines in cKO animals compared to control mice (t(8) = 3.82, *p* = 0.006; Fig. 9C). Further analysis revealed an altered distribution in spine types between control and cKO mice. Ventral CA1 pyramidal cells in cKO mice have a greater proportion of thin spines (30% in control versus 45% in cKO; *p* = 0.08), and a lower proportion of mushroom spines (61% control versus 49% cKO; p = 0.11) (Fig. 9B,D). These observations were made in basilar dendrites. No other significant changes in other parameters such as dendritic length and complexity, or in apical dendrites were observed (data not shown). These differences in spine types may influence synaptic activities, as mushroom spines tend to contain large excitatory synapses while thin spines are more immature and contain smaller excitatory synapses. However, the spine density per 10-micron length was comparable between control and cKO animals (t(8) = 1.35, *p* = 0.11; Fig. 9E). Similarly, the total cell body area was also comparable between the two genotypes (Fig. 9F). No significant differences in total basilar dendritic length were observed (Fig. 9G) (Control: 806 ± 67 µm vs cKO: 1148 ± 184 µm; *p* = 0.11; (t(8) = 1.75, *p* = 0.12). Overall, these results suggest that a deficiency of dysbindin-1 in CaMKII cells impacts spine dynamics trending towards more immature states.

### Expression of NMDA receptor subunits

**Figure 10:**
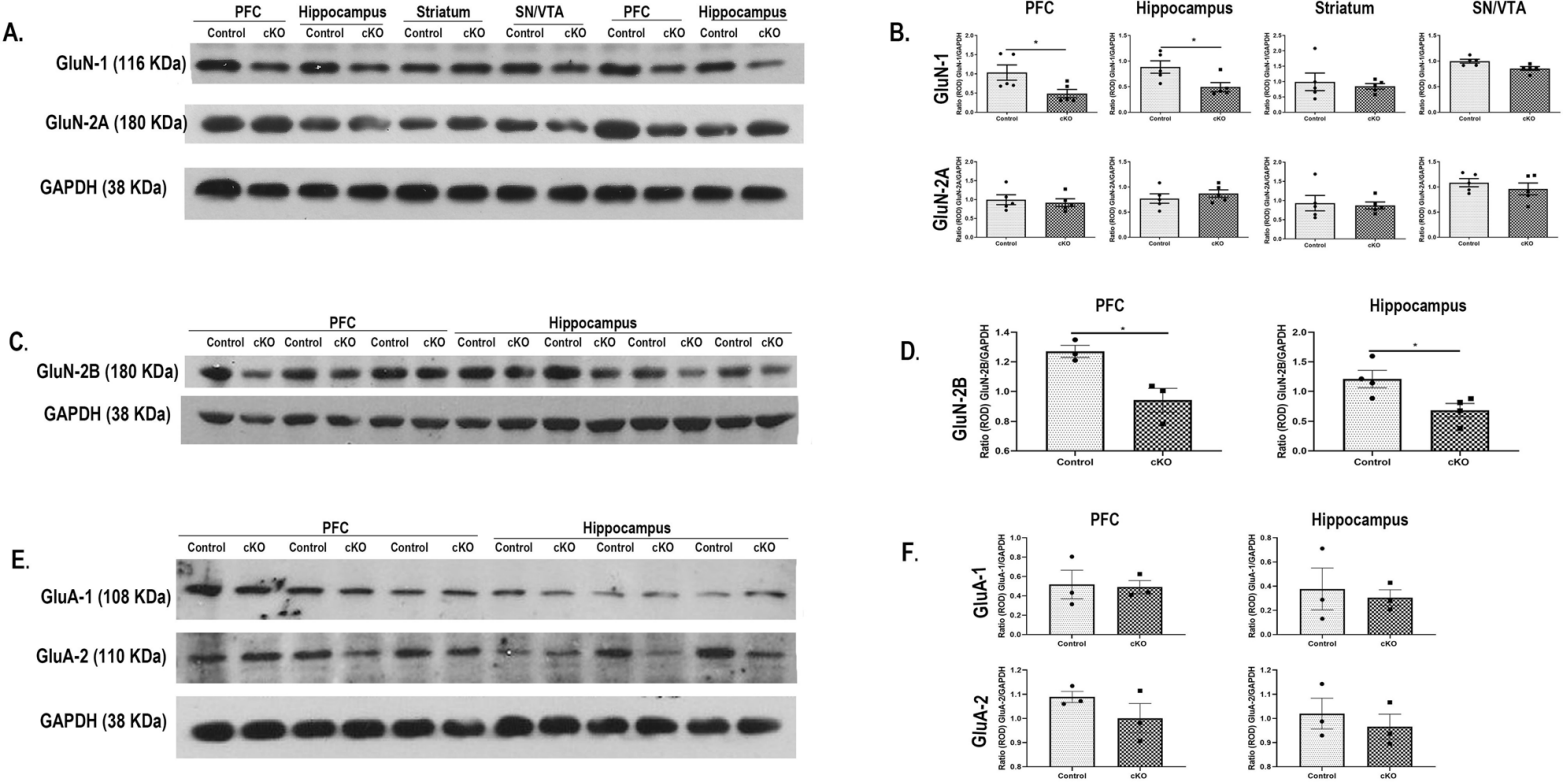
Decreased protein levels of total NMDA receptor subunits GluN1 and GluN2B protein in PFC and hippocampus of cKO mice. (**A**) Representative immunoblots of total GluN1, GluN2A and loading control GAPDH from two different control and cKO mice. (**B**) A significantly decreased level of total GluN1 was observed in the PFC and hippocampus brain regions of cKO animals compared to control mice. The levels of total GluN2A were not significantly different in any brain regions between control and cKO mice. (**C**) representative immunoblots of total GluN2B and loading control GAPDH from different control and cKO mice. (**D**) A significantly decreased level of total GluN2B was observed in the PFC and hippocampus brain regions of cKO animals compared to control mice. (**E**) representative immunoblots of AMPA receptor subunit GluA1, GluA2 and loading control GAPDH from control and cKO mice. (**F**) No significant differences in the levels of total GluA1 and GluA2 were observed in any brain regions of cKO animals compared to control mice. n: male control = 3–5, male cKO = 3–5. *: *p* < 0.05 vs Control.

Figure 10A shows representative Western blots of NMDA receptor subunit GluN1, GluN2A and a housekeeping protein GAPDH from PFC, hippocampus, Str and SN/VTA brain regions in control and cKO animals. Student’s t-test of normalized ROD showed a significantly decreased total GluN1 protein levels in the PFC (t(8) = 2.42, *p* = 0.041) and hippocampus (t(8) = 2.62, *p* = 0.030) in cKO animals compared to control animals. No significant differences in GluN1 levels were observed in other brain regions (Fig. 10B, top panel). The levels of GluN2A protein were not significantly altered in any brain region of cKO mice (Fig. 10B).

Figure 10C shows the representative western blots of NMDA receptor subunit GluN-2B and housekeeping protein GAPDH from PFC and hippocampus brain regions in control and cKO animals. Student’s *t*-test of normalized ROD showed a significantly decreased GluN-2B protein levels in the PFC (t(4) = 3.68, *p* = 0.02) and hippocampus (t(6) = 2.82, *p* = 0.030) in cKO animals compared to control animals (Fig. 10D).

Figure 10E shows the representative western blots of AMPA receptor subunit GluA-1, GluA-2 and housekeeping protein GAPDH from PFC and hippocampus brain regions of control and cKO animals. No significant differences in the levels of normalised GluA-1 or GluA-2 levels in any brain regions of cKO animals compared to controls were observed (Fig. 10F).

## Discussion

Our data suggest that a deficit of dysbindin-1 in putative excitatory CaMKII-expressing neurons leads to impairments in NMDAR-mediated behaviors, cognitive functions as well as alterations in hippocampal pyramidal cell morphology and excitatory transmission. As dysbindin-1 is expressed in a variety of cells, a comparison of our results with the global loss of function mutation in Sdy mice or conditional deletion in specific cells, e.g., dopamine neurons^13^ and astrocytic glial cells^17^ highlights similarities and differences in dysbindin-1 function in different cellular populations. For example, in contrast to increased spontaneous locomotor activity reported in Sdy mice^16^, we did not observe changes in spontaneous locomotion relative to controls in cKO mice. Interestingly, our previous results showed that a conditional deletion of dysbindin-1 in dopaminergic neurons of mice led to a hypolocomotor phenotype^13^. In addition, Sdy mice show deficits in basal PPI^43^ whereas we did not observe any significant changes in the basal PPI in the present study as well as in our previous study in dopamine cell-specific deletion of dysbindin-1^13^. Our findings are also in contrast to increased spontaneous locomotion and PPI deficits in mice with astrocytic deletion of dysbindin gene^17^. In addition, our study shows that the administration of MK801, an NMDA receptor antagonist, leads to an attenuated responses relative to controls. For example, MK801-induced locomotor hyperactivity and PPI deficits were significantly weaker in the cKO mice.

Similar to the observations made in Sdy global dysbindin-1 mutant mice^16,32^, our cKO mice also showed impaired spatial learning and memory in the Morris water maze, but no significant differences in anxiety-like behaviors in the EPM. Regarding social interactions, Sdy mice are reported to display reduced social interaction^35,44^. Another report suggests that a mutation in the dysbindin-1 gene, that reduces but not completely abolishes dysbindin-1 expression, results in no change in social vs object preference, but reduces social novelty recognition^45^. Our study in CaMKII cKO mice showing impaired social novelty memory without alteration in social preference appears to be in accord with Chang et al.^45^. Further, the absence of significant difference in one sample *t*-test in our cKO animals, in both SP and SNM tasks, further indicates sociability deficits as cKO failed to show significantly increased preferences for novel mice in both the tests. This decreased exploration of the novel mice is not due to the changes in locomotor activity or anxiety as there is no significant difference between control and cKO animals in the total exploration time of both the mice as well as in anxiety like behaviors as shown in EPM task. Interestingly, in our previous study in cKO of dysbindin-1 in DA neurons^13^, we did not observe significant alterations either in social recognition or spatial learning and memory. Our behavioral findings do not indicate significant sex differences between cKO and control animals which is in agreement with other studies in sdy mice^16^. Possibly, dysbindin-1 in excitatory neurons may not have a sexually dimorphic role.

Our results show a decreased expression of NMDAR subunits GluN-1 and GluN-2B with no significant changes in GluN-2A or AMPAR GluA-1 and GluA-2 subunits in the hippocampus and PFC of cKO mice. The attenuated effect of MK801 and cognitive deficits in CaMKII cKO mice may be related to the reduced expression of GluN1 we observed in the PFC and hippocampus^37^. Loss of dysbindin-1 has also been previously associated with altered NMDA-receptor subunit expression, depending on the region. For example, in the Sdy null mutant mice, GluN1 expression was comparable in the hippocampus relative to controls, but it was reduced in the frontal cortex. Further, although Papaleo and Weinberger^46^ reported that total GluN-2A levels was similar in sdy and control mice as we have observed in the present study, they found increased GluN-2A surface expression in both the frontal cortex and hippocampus. We believe that our data are indicative of a subtle hypofunctionality of NMDA receptors due to the dysbindin-1 deficit in excitatory neurons which is also suggested by our electrophysiological and morphological studies.

We found a reduction in the frequency of sEPSCs, but an increased amplitude in the CA1 pyramidal neurons of the ventral hippocampus in cKO mice. In contrast, no significant changes in sIPSCs were observed. Previous studies in Sdy mutants on DBA/2 J background also found a reduction in miniature EPSCs, but an increase in quantal size in hippocampal pyramidal cells^47^. Thus, our spontaneous input changes are in parallel to these previously reported changes in miniature synaptic currents. Further experiments would be required to elucidate the mechanisms of changes. Several experiments suggest that dysbindin-1 loss affects both presynaptic and postsynaptic terminals^48,49^. Although we observed a decrease in presynaptic function (reduced sEPSC frequency) and postsynaptic structures (changes in spine number and altered distribution), we did not find a reduction in the expression of GluA1 and GluA2. This may be due to the increase in AMPAR expression within each synapse after dysbindin knockdown, which is supported by an increase in sEPCS amplitude. However, the overall levels of AMPAR subunit expression in our cKO mice remain similar to control condition.

The mechanism by which loss of dysbindin-1 leads to decreased GluN1 and GluN2B protein levels, while decreasing spontaneous excitatory input frequency that are mediated by AMPA receptors, is not fully understood at this time, but may involve dysbindin’s role in regulating protein trafficking and synaptic function^47,50^. Dysbindin-1 is known to interact with proteins involved in vesicular trafficking and neurotransmitter release. It is possible that dysbindin-1 loss disrupts these interactions, leading to alterations in the trafficking and/or stability of NMDA receptor subunits GluN1 and GluN2B at the synapse. Further research is needed to elucidate the precise molecular mechanisms underlying these effects.

While we did not characterize presynaptic changes, we examined dendritic spine density and found an increased proportion of immature spines in cKO mice. This is in line with previous data showing that reduced dysbindin-1 expression using shRNA in cultured hippocampal cells is associated with reduced dendritic spine maturation^40^. Whether altered activity results in altered spine dynamics or altered cytoarchitectural changes produces changes in functional excitatory inputs is worth investigating. Given that dysbindin-1 associates with the WAVE2 complex involved in dendritic spine formation^40^, the loss of the protein may have resulted in improper spine formation resulting in immature spines. These immature spines translate functionally to a reduction in frequency of excitatory inputs by reducing the number of available fully functional postsynaptic sites. The increase in amplitude may in turn reflect the role of dysbindin-1 in kiss-and-run exocytosis of presynaptic vesicles^47^.

Overall, our results demonstrate that a deficit of dysbindin-1 in CaMKII-expressing cells produces cognitive and behavioral deficits that may be mediated by altered dendritic morphology, excitatory synaptic transmission, and NMDA receptor subunit expression. These studies with conditional deletion of dysbindin-1 in excitatory cells also suggest that cellular variations in dysbindin-1 expression may contribute to varied expression in behavioral outcomes. Finally, we identify the nuanced manner in which dysbindin-1 loss may impact dendritic spine morphology and excitatory transmission, including increased proportion of immature spines and increased frequency but decreased amplitude of excitatory postsynaptic currents. As dysbindin-1 is a potential candidate risk gene for schizophrenia and cognitive impairments^5^, our data may help better understand the contribution of this gene in the pathogenesis of the disorder.

### Supplementary Information

Supplementary Information.

## Data Availability

All processed data are in the figures of the manuscript. All raw data are available from the corresponding authors upon request.
