# Supplementary material for: Loss of dysbindin-1 in excitatory neurons in mice impacts NMDAR-dependent behaviors, neuronal morphology and synaptic transmission in the ventral hippocampus
Source: Sci Rep. 2024 Jul 2;14:15239. doi: 10.1038/s41598-024-65566-4 (PMC11219769; doi:10.1038/s41598-024-65566-4)

Loss of dysbindin-1 in excitatory neurons in mice impacts NMDAR-dependent behaviors, neuronal morphology and synaptic transmission in the ventral hippocampus.

Sanjeev K. Bhardwaj, Moushumi Nath, Tak Pan Wong and Lalit K. Srivastava

Supplementary Figure-1A: Raw gel blots images of Dysbindin-1 and CaMKII genotyping.

(Cropped lanes from these raw blots are presented in manuscript (Bhardwaj et al) as Figure-1A.

Floxed Dysbindin Genotyping

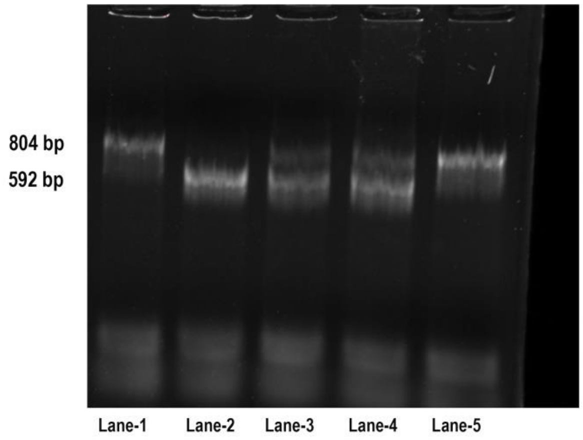

CamKII-Cre Genotyping

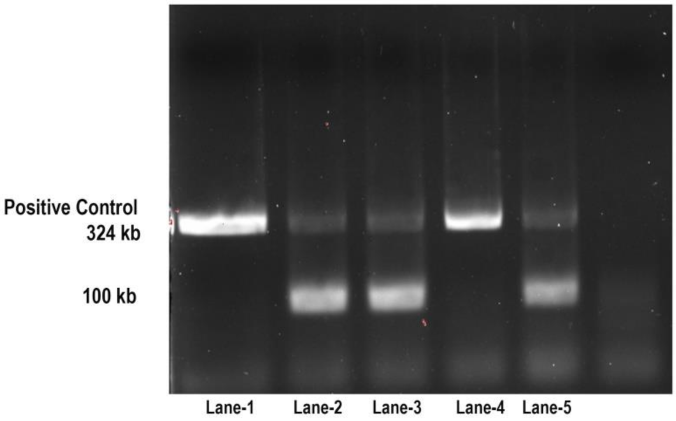

Supplementary Figure-2: Raw western blot images of Dysbindin-1 (2A) and housekeeping protein α-Tubulin (2B) in SN/VTA, Hippocampus, PFC and striatum (STR) brain regions of control (c) and cKO animals.

(Cropped bands from these raw gel blots are presented in Manuscript (Bhardwaj et al as Figure-1B)

2A

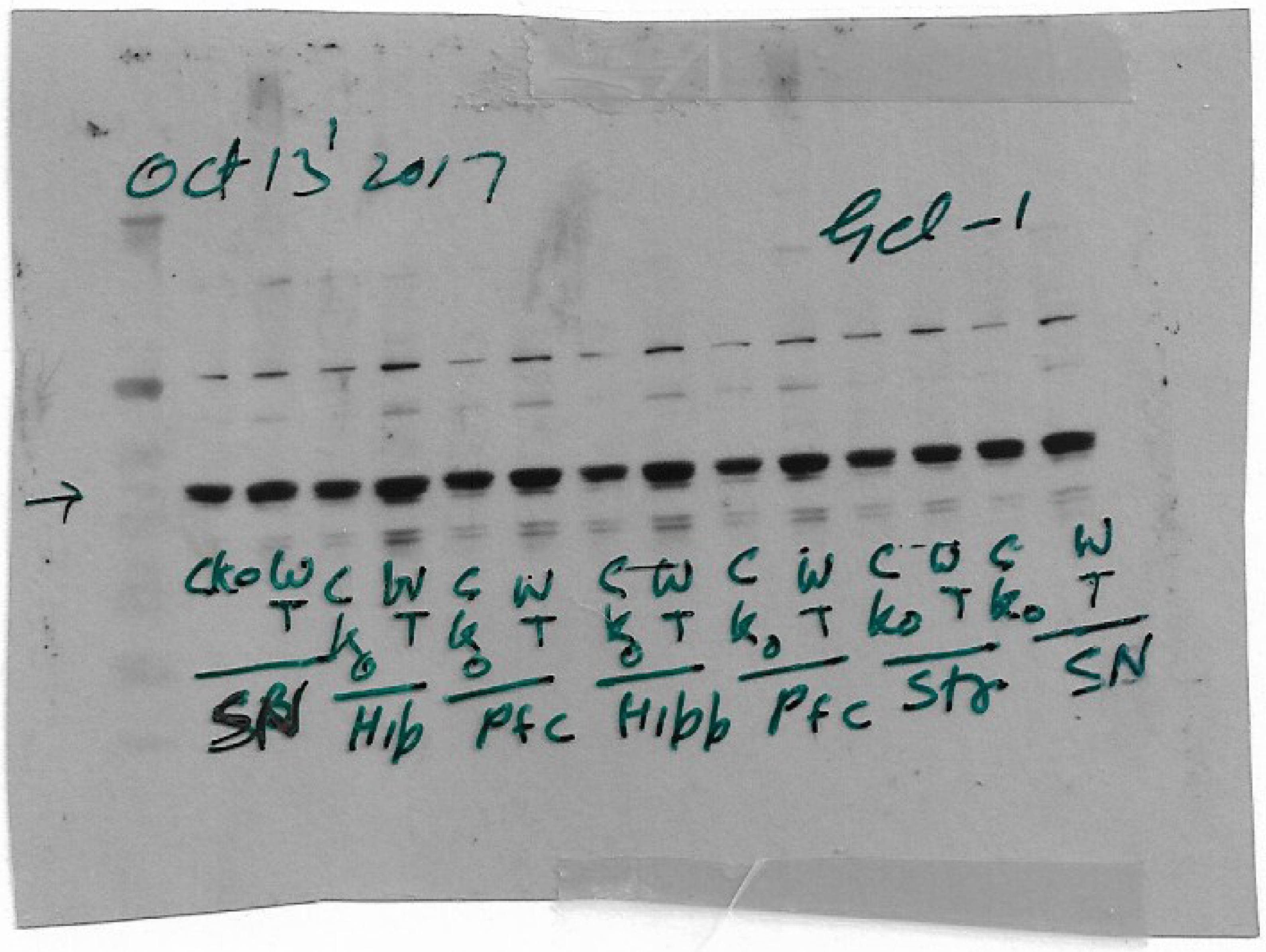

2B

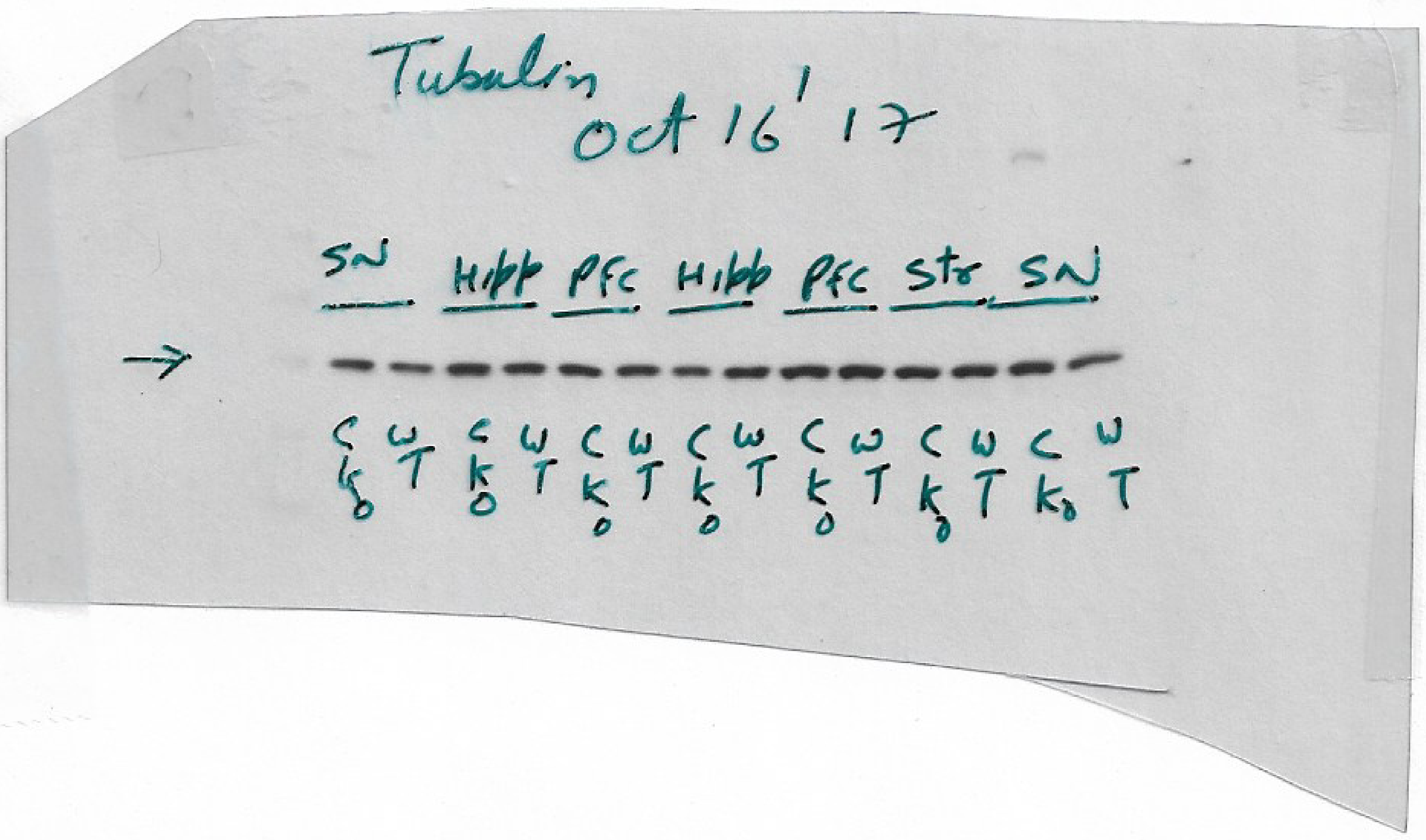

Supplementary Figure-3: Raw western blot images of NMDA receptor subunits NR1 (2A), NR2A (2B) and housekeeping protein GAPDH (2C) in PFC, Hippocampus, striatum (STR) and SN/VTA brain regions of control (c) and cKO animals.

(Cropped bands from these raw gel blots are presented in Manuscript (Bhardwaj et al as Figure-10A)

3A

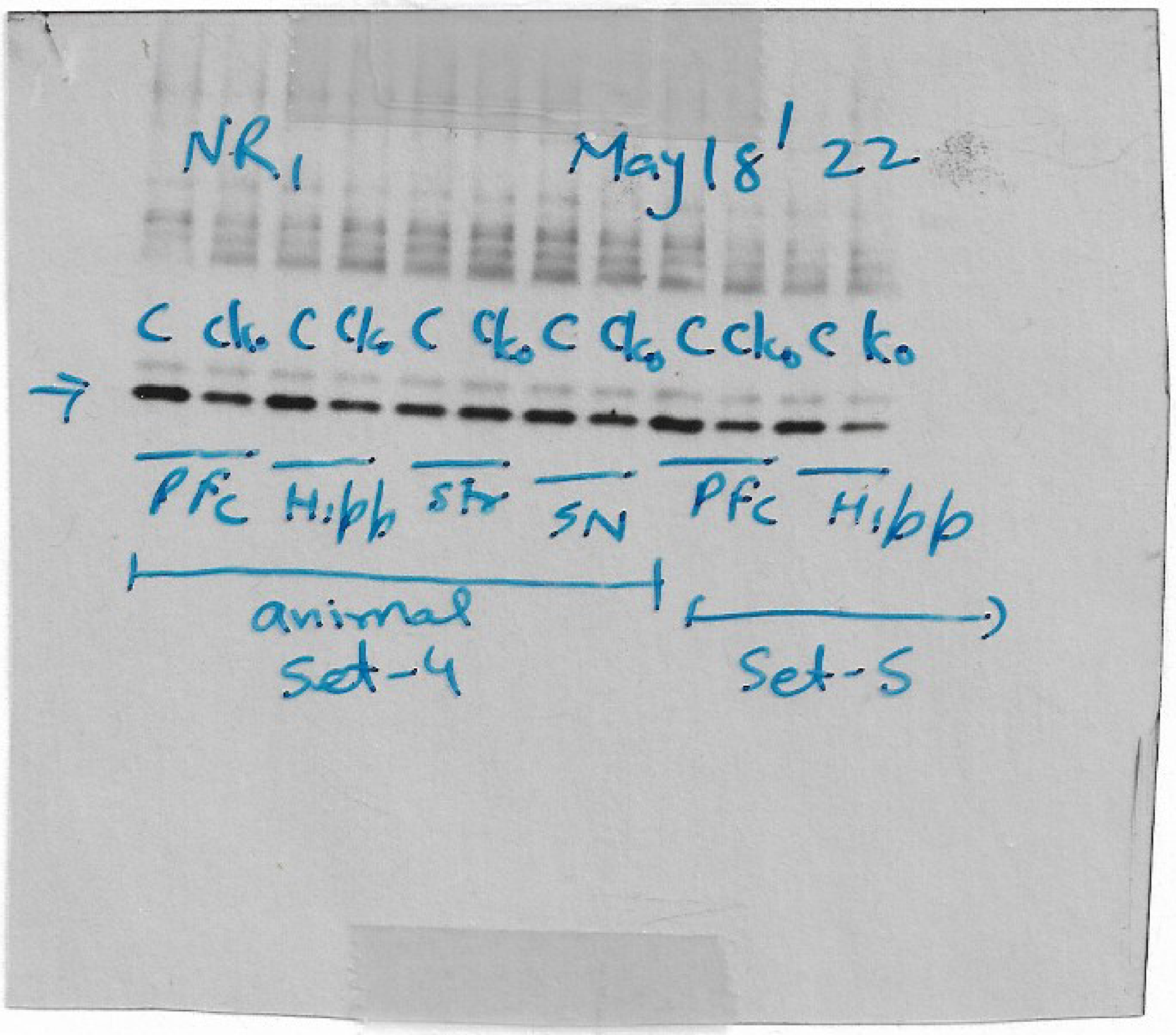

3B

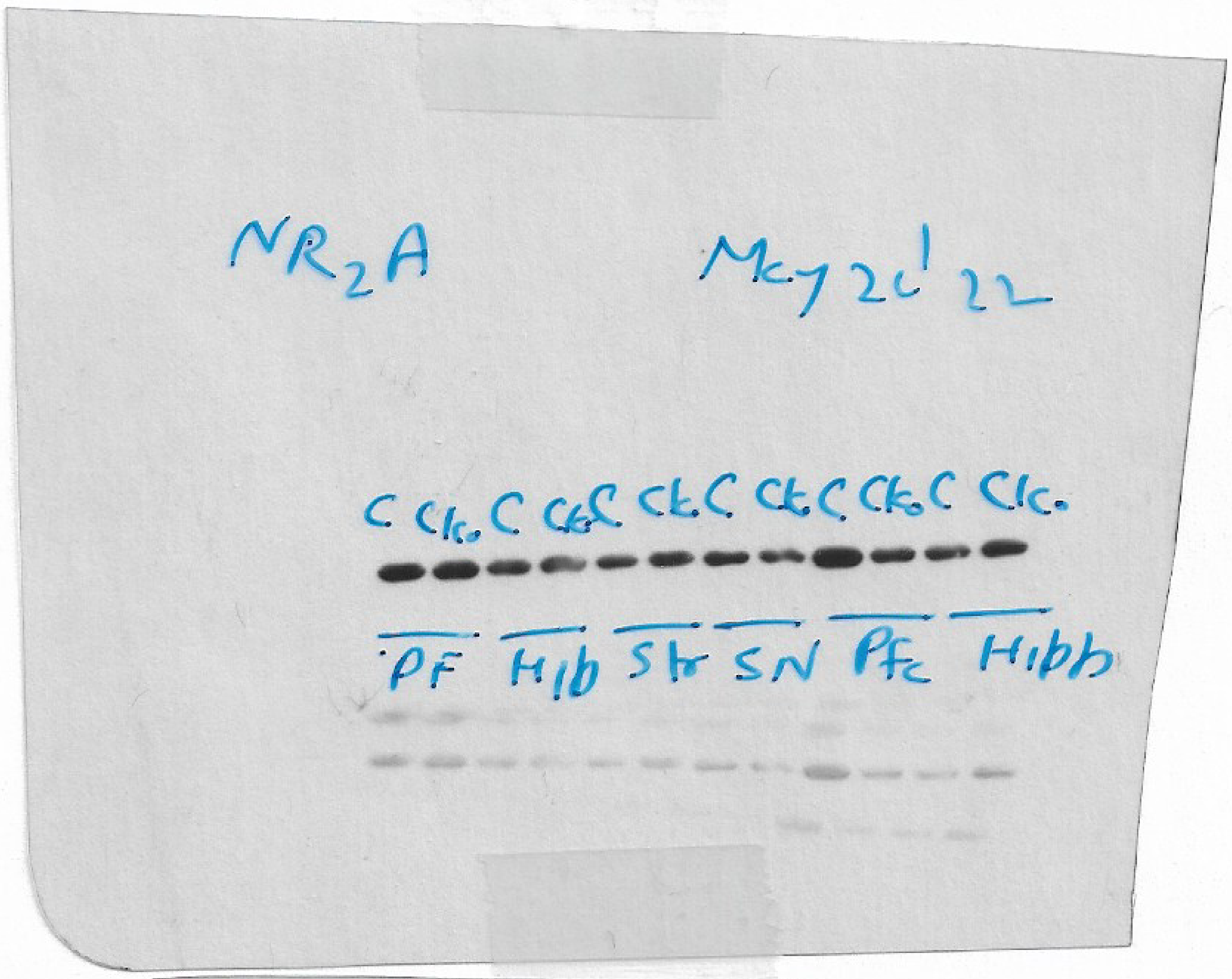

3C

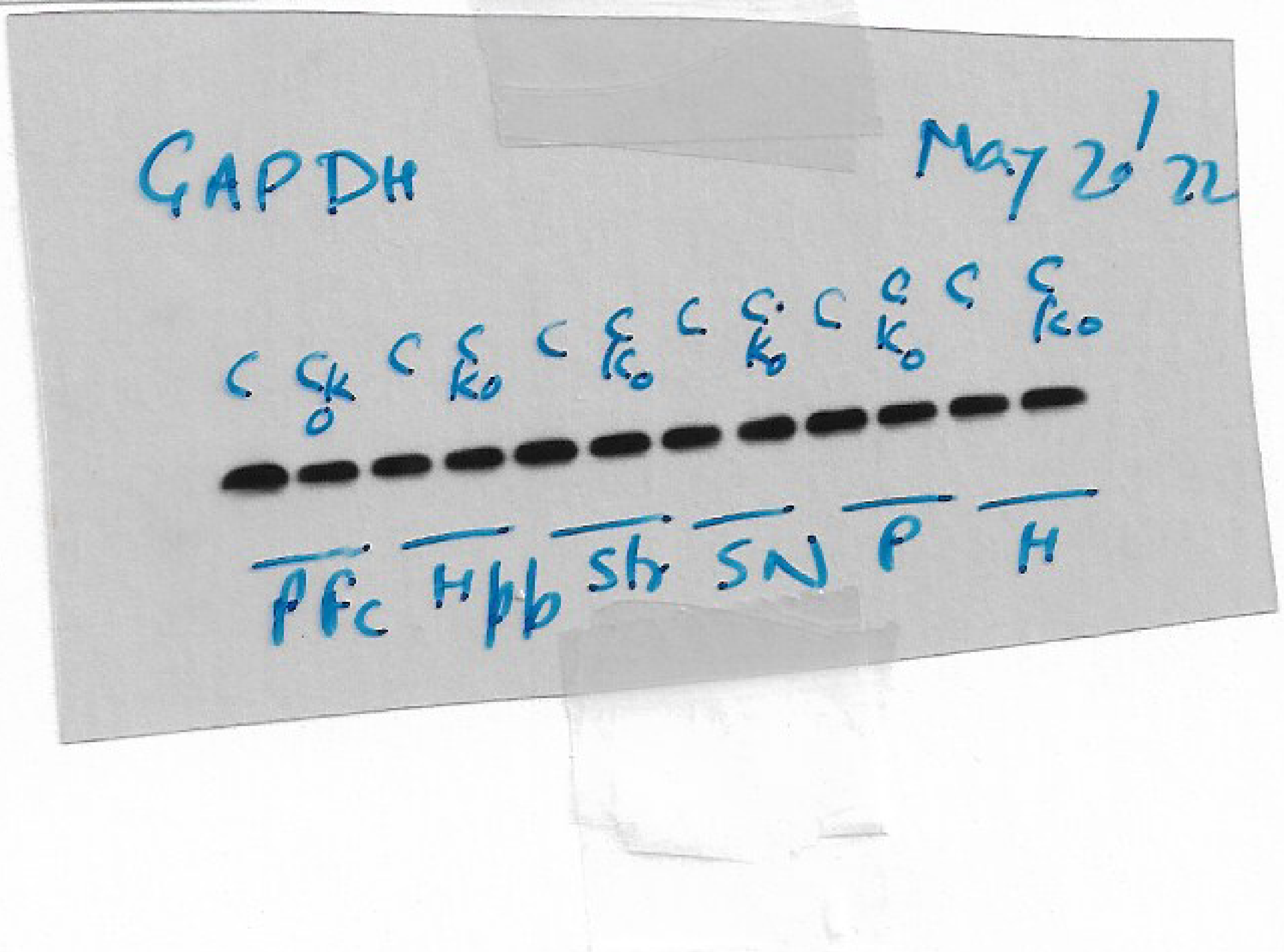

Supplementary Figure-4: Raw western blot images of GluN-2B (2A) and housekeeping protein GAPDH (2B) in PFC and Hippocampus brain regions of control and cKO animals.

(Cropped bands from these raw gel blots are presented in Manuscript (Bhardwaj et al as Figure-11A)

2A

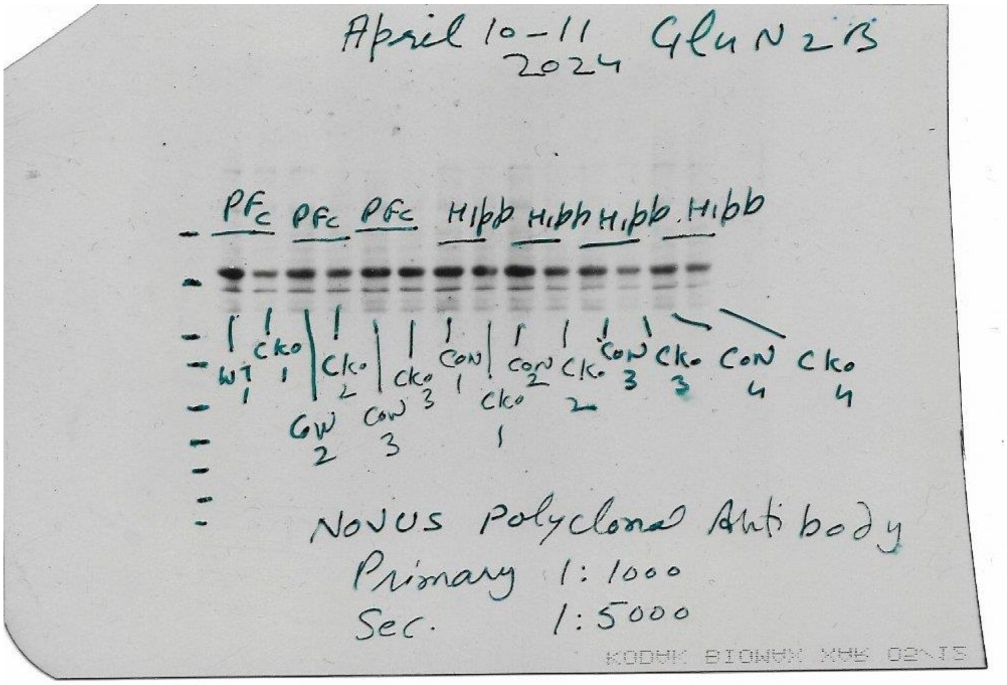

2B

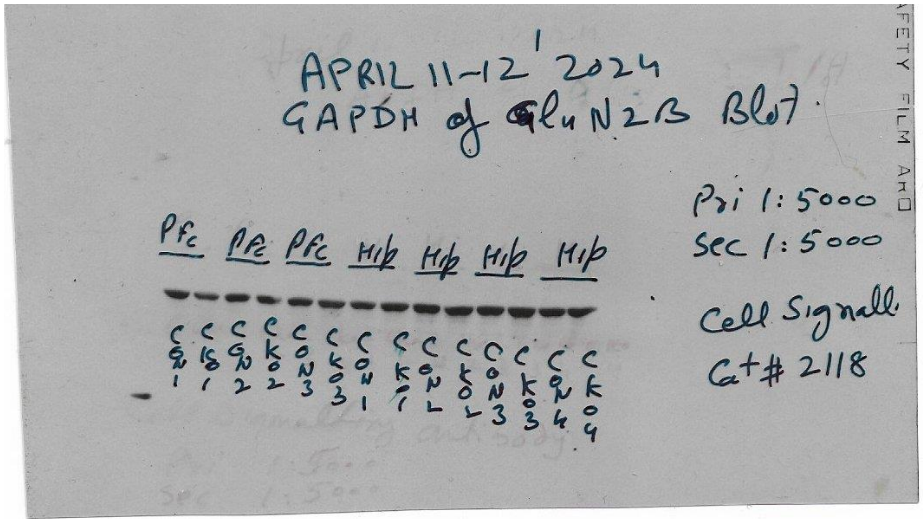

Supplementary Figure-5: Raw western blot images of AMPA receptor subunits GlUR1 (5A), GluR2 (5B) and housekeeping protein GAPDH (5C) in PFC and Hippocampus brain regions of control and cKO animals.

(Cropped bands from these raw gel blots are presented in Manuscript (Bhardwaj et al as Figure-12A)

3A

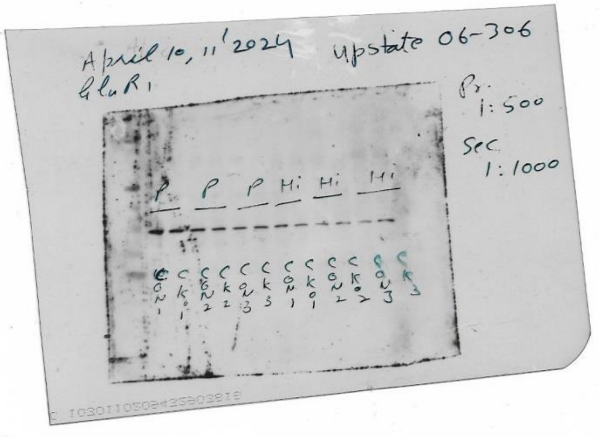

3B

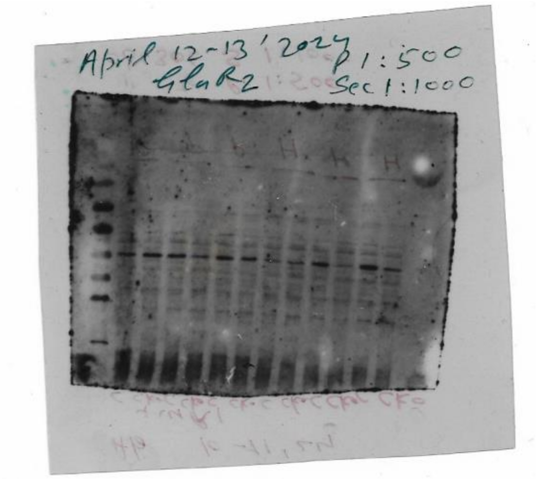

3C

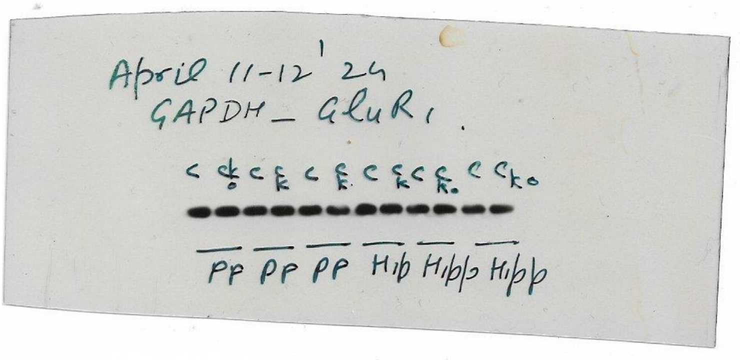

Supplement: Supplementary file 1 — Supplementary Information. [file 41598_2024_65566_MOESM1_ESM.pdf]
